# Internet of Bikes: A DTN Protocol with Data Aggregation for Urban Data Collection

**DOI:** 10.3390/s18092819

**Published:** 2018-08-27

**Authors:** Yosra Zguira, Hervé Rivano, Aref Meddeb

**Affiliations:** 1 Telecommunications Department, University of Lyon, INSA Lyon, Inria, CITI F-69621 Villeurbanne, France; Herve.Rivano@inria.fr; 2 NOCCS Laboratory Higher Institute of Computer Science and Communication Technologies, ISITCom, University of Sousse Sahloul, Sousse 4054, Tunisia; 3 NOCCS Laboratory, National Engineering School, ENISO, University of Sousse, Sousse 4054, Tunisia; Aref.Meddeb@infcom.rnu.tn

**Keywords:** internet of bikes, smart cities, internet of things, delay tolerant networks, wireless sensor networks, data aggregation, data collection, low-power long-range technology, LoRa/LoRaWAN

## Abstract

Intelligent Transport Systems (ITS) are an essential part of the global world. They play a substantial role for facing many issues such as traffic jams, high accident rates, unhealthy lifestyles, air pollution, etc. Public bike sharing system is one part of ITS and can be used to collect data from mobiles devices. In this paper, we propose an efficient, “Internet of Bikes”, IoB-DTN routing protocol based on data aggregation which applies the Delay Tolerant Network (DTN) paradigm to Internet of Things (IoT) applications running data collection on urban bike sharing system based sensor network. We propose and evaluate three variants of IoB-DTN: IoB based on spatial aggregation (IoB-SA), IoB based on temporal aggregation (IoB-TA) and IoB based on spatiotemporal aggregation (IoB-STA). The simulation results show that the three variants offer the best performances regarding several metrics, comparing to IoB-DTN without aggregation and the low-power long-range technology, LoRa type. In an urban application, the choice of the type of which variant of IoB should be used depends on the sensed values.

## 1. Introduction

The urban population of the world has grown quickly since 1950, from 746 million to 3.9 billion in 2014. The United Nations Population Division shows that, from 1950 to 2050, the share of the world’s population living in urban areas will grow from 30% to 70% [1]. This migration from rural areas to urban areas has been going on in developing countries, thus increasing the concentration in cities. The large scale urbanization results in enormous negative effects such as unhealthy lifestyle caused by smoke and dust, air pollution such as carbon emission, traffic congestion, etc. Biking has emerged as one of the major alternative transportation modes thanks to its flexibility, low cost and the benefits bikes provide such as a positive effect on the overall health [2]. Therefore, there is a worldwide trend to develop urban biking. In particular, bike sharing systems have been growing tremendous for a decade, covering more that 1000 cities with more than a million bicycles [3,4,5]. Consequently, several digital services have been developed to assist bikers and enhance their urban experience. Most currently running services are smartphone-based and are comparable to crowdsourcing/geolocalized applications. Following the trend of connected vehicle, the development of “smart bikes” arose, with integrated sensors and communication capabilities. The data they sense, analyze, collect, control and communicate can be useful for citizens, bike sharing system operators and municipalities.

In this paper, we consider a smart bicycle sharing system that senses and collects data. Bicycles are cheap and human powered, thus the integrated sensors and communication devices need to be low cost and use low power. They hence have a small amount of memory and computing power and are, overall, low end moving Internet of Thing (IoT) devices. We are interested in networking protocols that can efficiently support data collection, converge-cast, and application in these settings.

Mobile ad-hoc protocols were designed few decades ago with similar constraints. Nevertheless, most of these routing protocols suppose that the network is fully connected tolerating only short duty cycles for energy savings. At least an end-to-end path has to be available, computed a priori or on demand, each time a packet is sent. In practice, this assumption is hardly true in urban scenarios. Besides, the more dynamic is the network topology, the higher is the cost of signaling in these protocols, up to a point where the large majority of energy is spent in control and not payload transmission [6].

The Delay Tolerant Network (DTN) paradigm is an attempt to cope with highly dynamic topologies [7]. Tightly related to opportunistic communications, it is designed for intermittently connected networks facing long duration of partitioning or frequent movement. The core principle of DTN routing protocols is store-carry-and-forward: packets are stored in a buffer of a relay node, carried by it, and forwarded as soon as the “next link” is available. The main complexity of DTN routing compared to ad-hoc routing is then to decide which “next link” to use with an additional temporal dimension.

This paper is an extended paper of [8,9]. The main contribution of this paper is the design and evaluation of “IoB-DTN” (Internet of Bikes Delay Tolerant Network protocol), a data collection DTN-like protocol applied to a network of low cost, constrained and mobile IoT devices. The data sensed by the bicycles are relayed in a store-carry-and-forward manner and collected by a set of equivalent sinks. More precisely, IoB-DTN can be seen as a “lightweight” version of various flooding n-copy DTN protocols [10], optimized for IoT devices with several routing features removed for there are useless in converge-cast traffic. We compare this multi-hop approach with Low-Power wide-area network (LPWan, e.g., LoRa) technologies. LPWANs have been specifically designed for large scale coverage and could address the specific constraints of our settings [11]. We show that it is however not necessarily the best solution.

In particular, our second main contribution is to evaluate the potential gain provided by data aggregation mechanisms that leverage the relaying of data among bikes. Data aggregation approach is a vast research domain of wireless sensor networks [12]. Combining several data from potentially different sensors into a single packet reduces the number of actual data transmissions, hence saves energy and improves on the network throughput. Depending on whether it can alter the data, there might be a trade off between the intensity of aggregation and the data fidelity. We propose three variants of IoB-DTN: IoB based on spatial aggregation (IoB-SA), IoB based on temporal aggregation (IoB-TA) and IoB based on spatiotemporal aggregation (IoB-STA).

Through extensive evaluation by simulating real world data based urban scenarios, we derive the following engineering insights.

- Sharing packets among bikes improves on the performances, but there is no need to flood the network.
- Nevertheless, the buffer management policy of each bike has to give priority to its own packets over the relayed ones to obtain better delivery rates.
- There is an obvious trade-off between delivery ratio and average delivery delay.
- Long range low power technologies, e.g., LoRa or NB-IoT, are a serious challenger to multi-hop networks when energy is very constrained and throughput can be sacrificed.
- Taking advantage of the bike to bike transmissions with data aggregation mechanisms, in particular spatiotemporal aggregation, improves dramatically the performances of the protocol.

The rest of this paper is constructed as follows. Section 2 outlines the related work. Section 3 describes our scenario. Section 4 introduces IoB-DTN. Section 5 presents our simulation settings. The performance evaluation of IoB-DTN is depicted in Section 6. Section 7 presents the performance comparison between the multi-hop IoB-DTN and the low-power wide-area network technology, LoRa type. Section 8 dedicated to the performance improvement evaluation of IoB-DTN based on data aggregation approach. Section 9 concludes the paper.

## 2. Related Work

Our work extends a wealth of past works on communication based on four areas: public transport networks, DTN paradigm with the Internet of Things, long range technologies and data aggregation mechanism. In this section, we present previous works in each domain.

### 2.1. Communication Based on Public Transport Networks

Communication based on public transport network has been stressed in a number of recent works. Latora et al. [13] presented the first studies of networks based on urban transportation systems. They highlighted the importance of the use of real transportation networks in order to overcome many problems. The authors in [14] investigated the DakNet network. It offers a new solution based on low-cost communication for distant villages in Cambodia and India. In DakNet, public buses transmit the requests from kiosks in villages to a server to be treated and then forward the results to each one of them. In [15], the KioskNet is proposed as an intermediary network between kiosks in remote villages and gateways in which the data are carried by vehicles and buses. The authors also presented an itemized architecture that studies the issues related to the low-cost and reliable connectivity for distant kiosks. To cope with the disconnection of cars in vehicular networks, Zhao et al. [16] proposed three vehicle-assisted data delivery (VADD) protocols based on the store, carry and forward approach [17] denoted as L-VADD, D-VADD, and H-VADD. Each car carries the data packet until it encounters another vehicle and then it sends the data to it. From their experimental outcomes, their proposal Hybrid Probe (H-VADD) protocol is the best and performs better than existing solutions regarding to the delivery delay, protocol overhead and delivery rate. Burgess et al. [18] proposed MaxProp DTN routing protocol. It addresses the question of scheduling packets to be forwarded to neighbors nodes as well as deleting old packets when the buffer is full. The authors evaluated the proposal using a real network called UMassDieselNet that was deployed on 30 buses to collect data during two months. Their results show that Maxprop DTN routing protocol offers better performances than protocols knowing in advance the meetings schedule between nodes. The BusNet network is presented in [19]. It is a public transport system based sensor network designed to monitor air pollution as well as the road surface condition. It takes the advantage of deploying few mobile sensors mounted on buses instead of a large number of static ones. Thus, it reduces the issues related to the security in addition to the management maintenance.

Recently, bikes have been used as a city-based transport system for data collection applications. The first mobile sensing system based on bicycles was BikeNet project [20]. Sensors are mounted on cyclists bikes to collect quantitative data related to their journeys. BikeNet operates according two modes for data collection: opportunistic communications with gateways and real-time communications with mobile phones of cyclists to forward the sensed data. In [21], the authors designed the u-framework, a web framework for a ubiquitous sensor network to support application development using the database management system uTupleSpace. In their trial, they considered bikes with embedded sensors to collect data about environmental pollution in Tokyo, Japan.

In recent years, there are many public bike systems that can collect real-time data. In these schemes, sensors that are mounted on bicycles start collecting data when the bikes leave their bike stations such as schemes in Germany [22] and Netherlands [23]. In the literature, many studies have focused on analyzing the movement of bicycles in public hire systems such as the public cycle hire systems in Lyon [24], Barcelona [25], and London [26].

### 2.2. Communication Based on DTN Paradigm with the Internet of Things

In previous years, several researchers have focused on the application of the Delay Tolerant Networking paradigm to the IoT field [27]. Wirtz et al. [28] discussed the concept of “Challenged IoT” in which Internet disconnection between mobile users and smart devices is established. They proposed DISCO (Direct Interaction with Smart Challenged Objects) system enabling local and autonomous communications of smart objects. In [29], Al-Turjman et al. introduced an optimized delay-tolerant approach for Integrated RFID-Sensor Networks called DIRSN. It represents a new framework for integrated RSNs in the IoT field for data routing. The authors in [30] investigated the case of data collection with low energy consumption where the connectivity is frequently intermittent. They proposed an architecture to associate an M2M platform to Delay Tolerant Networks to gather data from mobile devices in an energy efficiency manner. Thanks to the wake-up system of sensors, their proposal reduces the energy consumption four times less.

Many researchers have focused on investigating DTN with IoT in the domain of delay-tolerant WSN that focus on routing algorithms [31]. Most of the proposal works do not use standard protocols, while they propose approaches dedicated to targeted applications or sensors such as studies on underwater sensor networks.

### 2.3. Communication Based on Long Range Technologies

In the last few years, new technologies have been designed to improve communication. Next generation networks (5G) will be launched on the market by 2020. 5G improves the speed as well as it can facilitate the emergence of a huge IoT ecosystem in which networks can communicate billions of connected objects, thanks to a balanced compromise between speed, latency and cost.

Many low-power wide-area network (LPWAN) technologies have also been deployed. LPWA networks are characterized by having several forms as well as different market approach and technology stack. The most used LPWAN technologies are LoRaWAN, Sigfox and Weightless. LoRa/LoRaWAN technology (LoRa: https://www.lora-alliance.org/) was developed by the start-up Cycleo in 2009 in Grenoble city, France and was purchased by Semtech (Newbury Park, CA, USA) in 2012. In 2015, LoRa technology was standardized by LoRa-Alliance and was deployed in 42 countries. Its architecture is based on chirp spread spectrum modulation which uses the same low power characteristics as FSK modulation, whereas immensely raises the communication range. The LoRa physical layer operates on the 868 MHz in Europe, 433 MHz in Asia or 915 MHz in North America ISM bands.

In 2010, the Sigfox technology (Sigfox: https://www.sigfox.com/en) was developed by the start-up Sigfox in Toulouse, France. It is characterized by the application of a technique based on ultra-narrowband IoT communications designed to support IoT deployments over long range communications. It operates in the 869 MHz in Europe and 915 MHz in North America bands. The signal of Sigfox is narrowband using channel bandwidths lower than 1 KHz and forwarding a maximum payload size of 12 Bytes.

With regard to Weightless technology (Weightless: https://www.weightless.org/), the Weightless Special Interest Group has developed three open standards: *Weightless-W*, *Weightless-N* and *Weightless-P*. Weightless-W is based on narrow-band FDMA channels with Time Division Duplex between uplink and downlink. It is designed to be performed in TV white-spaces (470–790 MHz). Weightless-N is based on the ultra-narrow-band technology and it gives only uplink communication. Weightless-P offers ultra-high performance LPWAN connectivity.

### 2.4. Communication Based on Data Aggregation Mechanism

In recent years, several works based on data aggregation in wireless sensor networks have been carried out [32]. According studied works in the literature, there are two categories of data aggregation: aggregation structures and aggregation functions.

On the one hand, many works have focused on aggregation structures such as tree-based, cluster-based and backbone-based structures. The authors in [33] introduced a centralized algorithm and design a distributed protocol for building a tree routing structure with maximum lifetime. Kuo et al. [34] proposed a data aggregation tree (MECAT) which can minimize the total energy cost of data forwarding and have introduced a 2-approximation algorithm. They studied two issues: the first one without taking into account relay nodes and the second one with the consider of relay nodes having imperfect link quality. They proved for the first problem that every shortest path tree has an approximation ratio of 2. According to second case, the problem is proven to be NP-complete and a seven-approximation algorithm is proposed. The authors in [35] introduced a spatial clustering algorithm for sensor networks. It can construct a dominating set based on the information description performance of the dominators to perform the data aggregation. In [36], Sinha et al. proposed an efficient clustering protocol in wireless sensor network that offers significant energy savings. They performed data aggregation on the basis of entropy of the sensors. The clustering process is distributed and based on the sensed data category, independently of geographic positioning and distance measures. Xu et al. [37] proposed Data Quality Maximization (DQM) protocol based on a backbone that is composed of a set of gateways. The authors investigated a mobile sink moving along a fixed trajectory without stop to collect data. Their proposal protocol is based on predictability of the sink movement and selects the gateways adjacent to the predicted path of the sink. Cui et al. [38] proposed Similar-evolution Based Aggregation (Simba), a raw data-independent aggregation to consider the evolution of data rather than the raw data. The Simba proposal creates a group of isolated nodes which perform data aggregation, therefore reducing the energy consumption in the network.

On the other hand, several works have focused on aggregation functions. They represent the way to do aggregation. In [39], the authors presented an experimental study that uses the ARIMA model for the design of an energy efficient data collection in wireless sensor networks. Their proposal avoids sensor nodes to deliver redundant data, this can be predicted by the sink node. Lu et al. [40] proposed an A-ARMA method based on the forecasting by means of an ARMA model over moving average windows. The use of the A-ARMA technique reduces the computation in every sensor node and it does not have a pre-computation phases. In [41], the authors proposed Agnostic Aggregation (A2), a dynamic forecasting function. Their proposal can predict values with self-tuned model based on temporal aggregation. The authors in [42] presented the theory of compressive sampling methods.

## 3. Scenario Description

We investigated an ad-hoc network of bicycles. More specifically, we considered a smart bike sharing systems for data collection application. Each bicycle has embedded sensor and a 802.11p communication device. The bikes read their sensors, generate packets every second and store them in their buffers. All bicycle sharing stations are equipped with base stations that are connected to the Internet. They have a 802.11p interface and act as fixed sinks. In our scenario, a packet is relayed until it reaches one of the sinks. Only when a node is in communication range with base stations, it forwards all data stored in its buffer to them.

**Figure 1:**
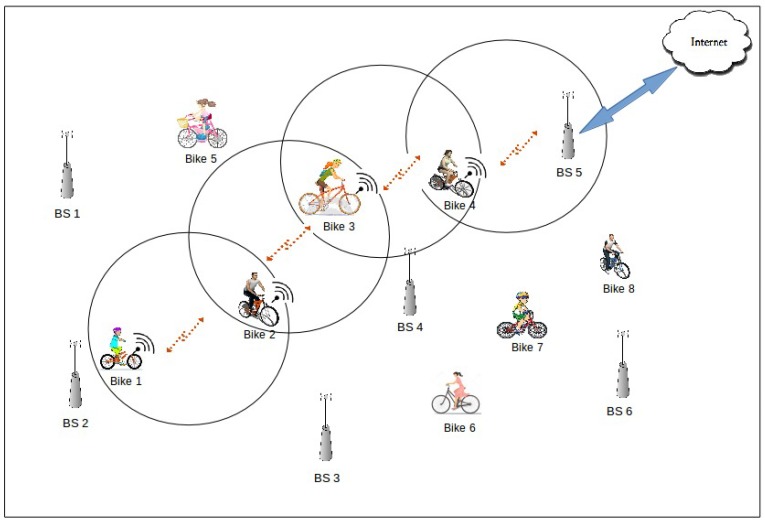
Illustration of our smart bike sharing system.

A simple scenario with eight bikes and six base stations is depicted in Figure 1. Bike 1 leaves Base Station 2 and starts generating data. After a certain period of time defined in seconds and when Bikes 1 and 2 are within communication range, they exchange data stored. Each bike stores the data in their buffers until it encounters a base station. Bike 4 lies in the area of Base Station 5, hence it forwards all data stored in its buffer. In this way, data are stored in the buffers of neighboring nodes until they reach the destination.

## 4. IoB-DTN: Internet of Bikes-DTN Protocol

This section introduces the IoB-DTN protocol. It is based on the DTN paradigm which is designed for networks that may lack continuous connectivity. To cope with the intermittent connection between bicycles, the store-carry-forward mechanism is applied [43]. The data are forwarded to intermediate nodes where they are kept and sent at a later time to another intermediate nodes or to the final destination.

Unlike the mobility models of other transport systems that are expected and occasional, our mobility pattern of our smart biking network is human created. From this perspective, IoB-DTN protocol is based on replication-based routing protocols, called flooding approaches, that do not require knowledge about the network topology to route data [44]. Flooding approaches rely mostly on replication. Multiple copies of each message are diffused to a set of nodes, called relays, with the purpose of maximizing the delivery probability of packets. Notably, IoB-DTN is a lightweight variant of Binary Spray and Wait DTN routing protocol [45] in which the number of copies sprayed in the network is limited. Hereby, it reduces energy consumption of nodes. Binary Spray and Wait protocol is an improvement over Spray and Wait [45]. The source of a message initially starts with L copies; any node that has at least two copies and encounters another node having no copies forwards n/2 stored copies and keeps n/2 for itself. When it is left with only one copy, the last copy is transmitted to final destination.

The description in details of IoB-DTN protocol is given in Algorithm 1. In IoB-DTN, each node generates periodically a packet *P* with the readings. The packet *P* and its initial number of copies *N* are stored in the buffer if the buffer management policy used provides a slot. When the duty cycle is over, each node verifies the existence of a base station in its vicinity. If the node lies in the area of one or more base stations, it forwards all data stored in its buffer to only them. If not, it sends packets having more than one copy, their number of copies and its neighbors list to neighbors nodes. When receiving a packet, each node determines its position in the neighbors list of the source node in order to calculate the new number of copies $N^{\prime }$ to be kept. The received packet is then stored in the buffer with $N^{\prime }$, if it is at least equal to one, and an acknowledgement (ACK) is sent to the source node. At the reception of an ACK, each node deletes the corresponding packet if the sender node is a base station, alternatively it updates the number of copies *N*′.

**Algorithm 1** IoB-DTN protocol.
1:At each sensor reading period2:Generate a packet *p* with the readings3:**if** Buffer management provides a slot **then**4: Store $p\cup N^{0}$ in the buffer [$N^{0}$ copies of *p* are stored]5:
**end if**
6: 7:When duty cycle is over8:L← the list of neighbors9:**if** a base station is in L **then**10: Send all packets in buffer11:
**else**
12: **for all** packet p∪N in buffer **do**13:  **if** N (number of copies) > 1 **then**14:   Send p∪N∪L15:  **end if**16: **end for**17:
**end if**
18:Wait for next duty cycle19: 20:On reception of packet p∪N∪L21:pos← self position in L22:
$N^{\prime }\leftarrow \frac{N}{2^{pos+1}}$
23:**if** Buffer management provides a slot **and** $N^{\prime }\geq 1$ **then**24: Store $p\cup N^{\prime }$25: Send ACK for receiving $N^{\prime }$ copies of *p*26:
**else**
27: Packet is rejected, no ACK is sent28:
**end if**
29: 30:On reception of an ACK of *p* and $N^{\prime }$31:**if** sender node is a base station **then**32: Delete *p* from buffer33:
**else**
34: Update the number of copies of p:$N^{\prime \prime }\leftarrow N-N^{\prime }$35:
**end if**



## 5. Simulation Settings

**Figure 2:**
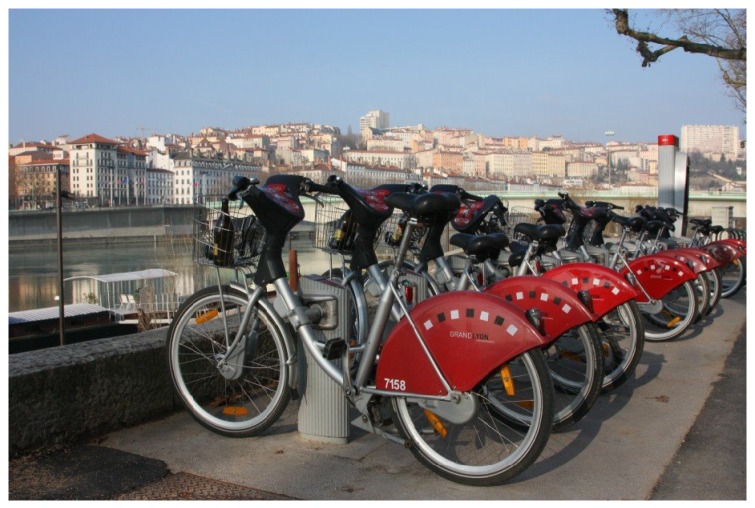
Vélo’v bicycle sharing system in Lyon.

IoB-DTN protocol was evaluated in the area of Lyon, France. It is in east central France belonging to the Auvergne-Rhône-Alpes region and is the second largest urban city of France. The self-service bicycles in Lyon is called Vélo’v (https://velov.grandlyon.com/) and it has been managed by JCDecaux since May 2005. The metropolis of Lyon has provided about 4000 bicycles, which are available 24/7, distributed in more than 300 bike stations scattered in the cities of Lyon and Villeurbanne. This mobility service offers the possibility to make local trips mainly in the urban areas. It allows citizens to rent a bike to move from one zone to another. One of the bike stations in Lyon is presented in Figure 2.

**Figure 3:**
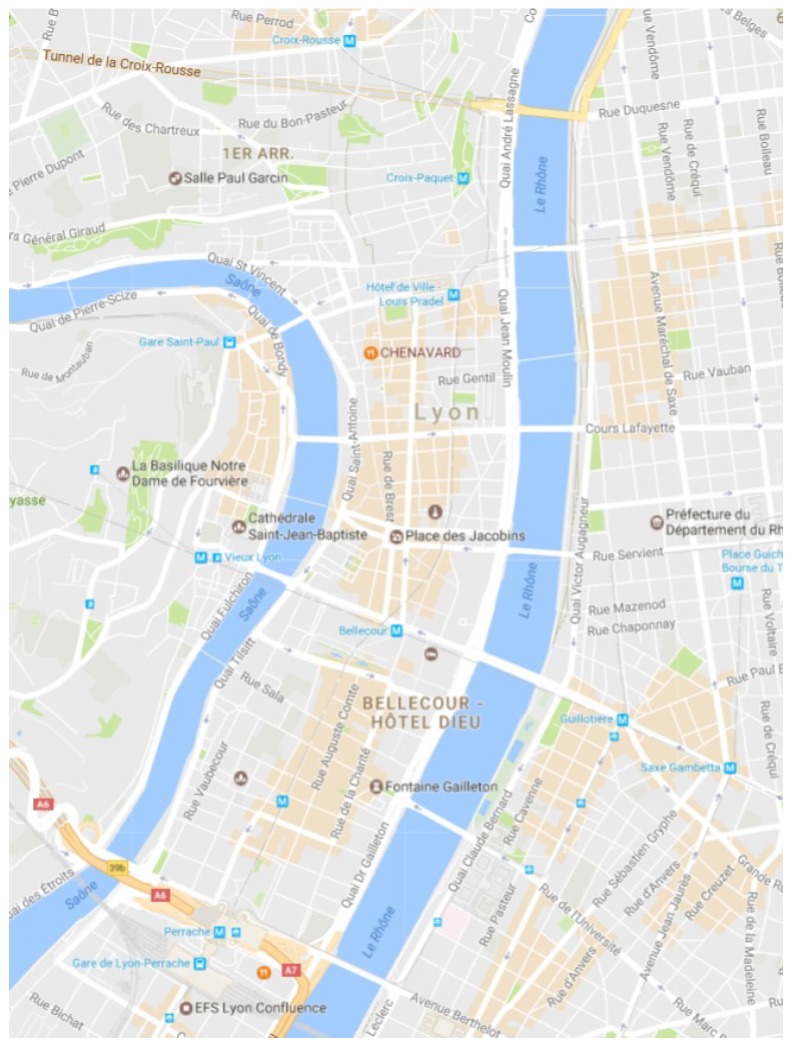
Considered area of Lyon.

The platform “Data Grand Lyon” provides open data regarding the Vélo’v bike stations (https://data.grandlyon.com/). These inputs are combined with the map of the city of Lyon that is generated from Open StreetMap (https://www.openstreetmap.org). The area considered for our simulations is in the city center of Lyon, as depicted in Figure 3. The fusion of those data is imported to the open source road traffic simulator “SUMO” [46]. It is designed to handle bicycle routes, road networks and obstacles. The open source framework for running vehicular network simulations “Veins” (http://veins.car2x.org/) is used to connect the road traffic simulator “SUMO” to the network simulator “OMNeT++” (https://omnetpp.org/) via a TCP socket. Veins includes a real radio propagation and 802.11p models.

**Figure 4:**
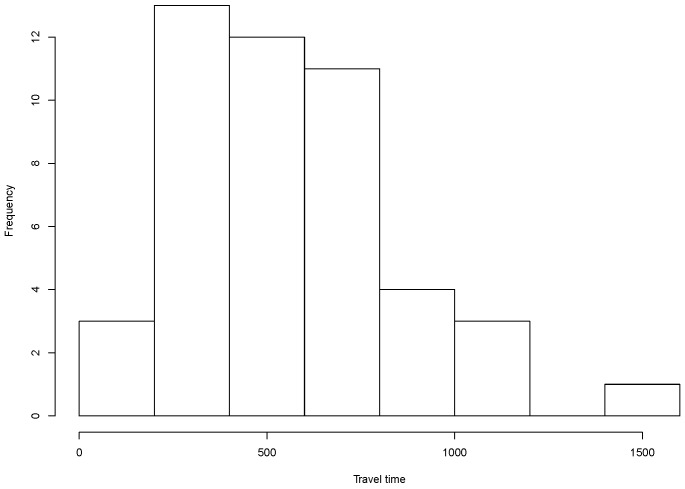
Bikes travel time in seconds.

In our simulated scenario, each mobile bike generates a data packet every second. The travel time of bikes corresponds to the trip duration of each bike in the simulation. The longest travel time of bicycles is 1418 s while the average one is about 550 s, as illustrated in the histogram in Figure 4.

We simulate four sets of parameters as shown in Table 1 by varying the buffer size and the duty cycle:

- **SB-SDC**: Small Buffer-Short Duty Cycle;
- **SB-LDC**: Small Buffer-Long Duty Cycle;
- **LB-SDC**: Large Buffer-Short Duty Cycle; and
- **LB-LDC**: Large Buffer-Long Duty Cycle.

It is interesting to note that the copies of a packet stored in a buffer are virtual. We just increment a counter and each packet occupies only one slot of the buffer. In other words, the buffer size is given as the number of slots in bytes. The duty cycle is a period defined in seconds, after which each node forwards the data packets stored in its buffer.

Table 2 summarizes the simulation configuration used for our scenario.

## 6. Performance Evaluation of IoB-DTN

In this section, we evaluate the performance of IoB-DTN protocol. First, we assess four buffer management policies. Then, we compare the impact of the number of copies spread in the network. Finally, we evaluate the impact of the variation of the transmission power of sensors.

### 6.1. Impact of Buffer Management Policies

The buffer management policy is an important parameter of IoB-DTN protocol. It is used to find a slot in the buffer when a packet is generated or received. If the buffer is not full, it provides the first free slot. If the buffer is full, it decides which packet should be kept and which packet should be discarded with the risk that no copy reaches the destination. We propose four management policies. When the buffer is full, they perform as follows:

1. **KONP: Keep Oldest No Priority:** If the buffer is full, the new generated packet or the received one is always deleted.
2. **NP: No Priority:** If the buffer is full, the new packet replaces the oldest one. This buffer policy is based on the concept that a packet spending a lot of time stored in a buffer is more likely to be sent to a neighbor node or to a base station.
3. **GPP: Generated Packet Priority:** This policy averts the cases in which received packets take up all the slots in the buffer by giving the priority to the self-generated packets. Thereby, if the buffer is full and a new packet is generated, it replaces the oldest received packet. Otherwise, if there are only generated packets stored in the buffer, it replaces the oldest one. All received packets are rejected.
4. **LC: Lesser Copy:** This policy is based on the number of copies parameter of each packet stored in the buffer. It considers that a packet having the fewest copies has a high probability to be transmitted to another neighbor node or to its destination. Thus, when the buffer is full, the packet having the fewest copies is rejected and replaced by the new one.

We compared the performance of the four buffer management policies cited above with IoB-DTN protocol when the number of copies of a packet is limited to 8. We evaluated two metrics: the loss rate and the delivery delay which is the time between the generation of a packet and its reception by a base station.

**Figure 5:**
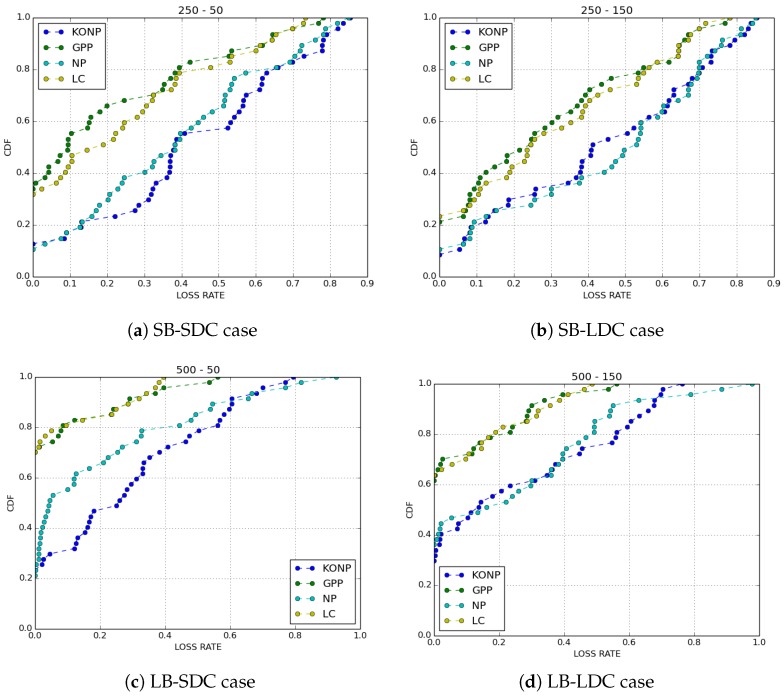
Loss rate.

Figure 5 shows the loss rates obtained for all cases. The first observation we make is that GPP and LC have better performances than KONP and NP buffer management policies. GPP policy provides better performances in all cases. Normally, forwarding duplicated packets in the network has less impact on the loss rate for GPP since it gives priority to the generated packets. Nevertheless, GPP performs better when the duty cycle is lower, thus showing that the redundancy used by the protocol offers robustness. LC has the same performance as GPP. As expected, by discarding packets having the fewest copies has a high probability to be arrived to a base station, hence rises the redundancy of the packets in the network. NP outperforms slightly KONP. It offers bad results comparing to GPP and LC since it drops oldest packets if they are generated or received. KONP is the worst one, in particular when the buffer size is small. This is because the generated packets saturate very quickly the buffer, thus all new packets are rejected.

**Figure 6:**
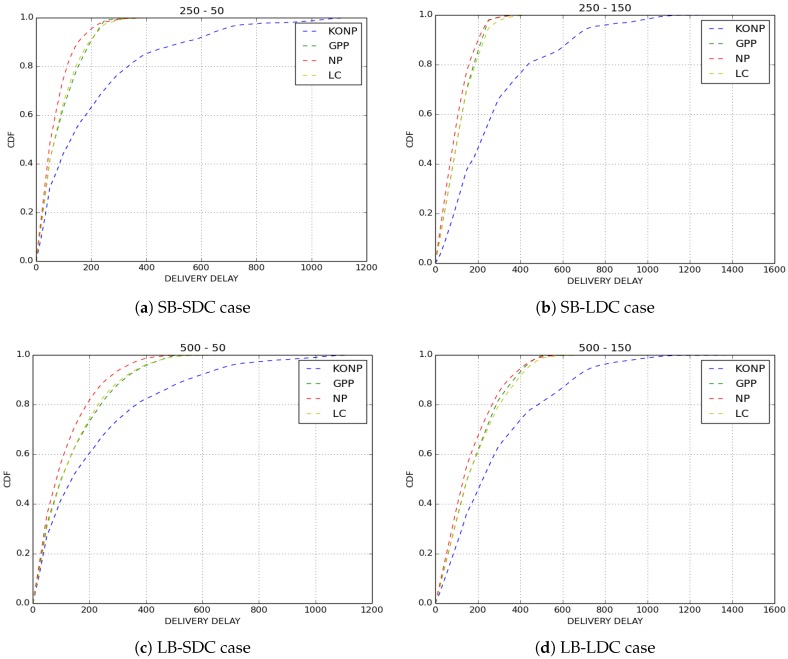
Delivery delay.

The delivery delays of received packets are depicted in Figure 6. We observe that, as expected, NP policy has the best results in terms of delivery delay since it drops old packets. KONP gives the worst performance. This is an evident outcome for a policy of which only old packets are forwarded. GPP and LC policies have the same performance as NP while giving a better loss rate. This means that there are older packets that are transmitted and the fact that they have similar delay distribution shows that they are sent very quickly.

From our results, we can classify two categories of policies: KONP and NP on the one side; andGPP and LC on the other side. It is clear from our evaluations that KONP is the worst policy as it has poor performance on loss rate and delivery delay. The performances of GPP outperforms slightly LC in all cases despite their different behaviors: GPP prioritizes own production against redundancy, whereas LC is based on redundancy to give place to new packets.

**Figure 7:**
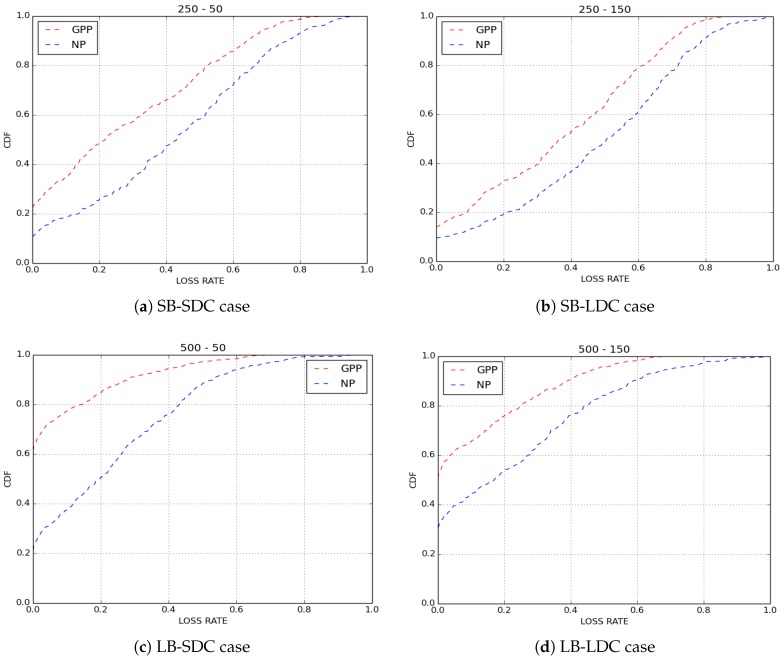
Average loss rate.

**Figure 8:**
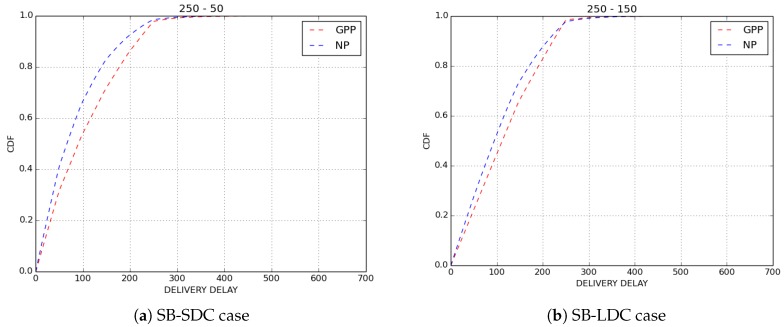

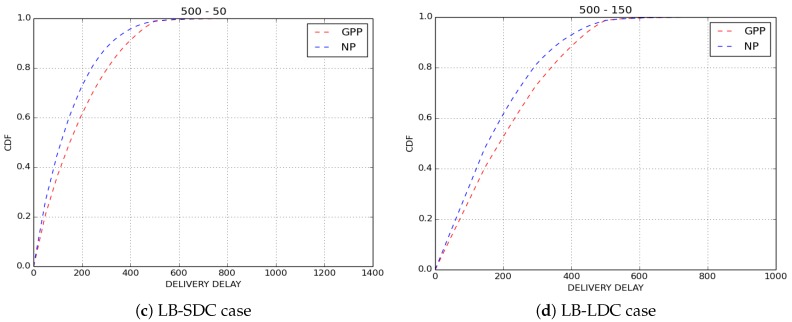
Average delivery delay.

For the purpose of confirmation of our results, we simulated ten scenarios for GPP and NP policies by varying the paths of bikes in each simulated scenario. The average findings obtained on loss rate are depicted in Figure 7 and results on delivery delays are shown in Figure 8. Clearly, we obtained the same results as those presented here.

In the rest of this paper, we consider GPP as the buffer management policy for all our simulations.

### 6.2. Impact of Number of Copies

The number of copies $N^{0}$, created during the generation of a packet, is a paramount parameter of IoB-DTN protocol. As discussed above, each node replicates and sends to the neighbors nodes only the packets having more than one copy in its buffer. Thus, the first neighbor node stores half the copies, the second one stores one fourth, the third one an eighth and so forth. The larger is $N^{0}$, the more redundancy is performed.

For our study, we evaluate three variants of IoB-DTN protocol:

- $N^{0}$ = 2: Two-Hop Relay protocol;
- $N^{0}$ = 8: Binary Spray and Wait protocol; and
- $N^{0}=\infty$: Epidemic Routing protocol.

In *Binary Spray and Wait* DTN protocol [45], the number of copies is limited to 8. By assigning the number of copies to 2, IoB-DTN gets the behavior of *Two-Hop Relay* DTN protocol [47]. The operation of this protocol is simple and works as follows: all packets are duplicated to only first encountered nodes, then the source node and the first encountered neighbor node will carry the duplicated packets until a base station comes in range. When $N^{0}=\infty$ (large value), IoB-DTN imitates the behavior of Epidemic Routing DTN protocol [17] which floods the network. Here, we are interested in comparing these three variants to evaluate the impact of the number of copies sprayed in the network. We only give a comparison between GPP, which provides better performances on loss rate and delivery delay, and NP, which offers the best delivery delay.

**Figure 9:**
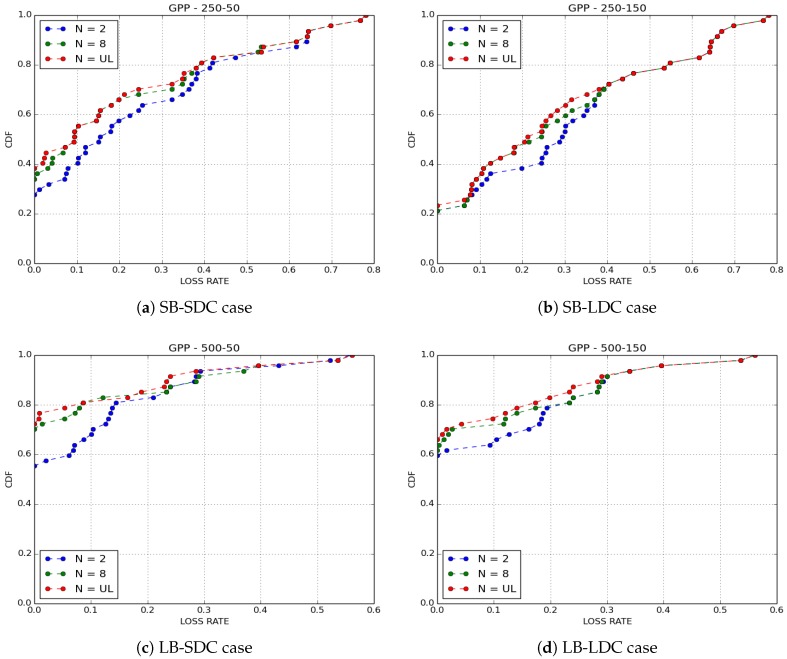
Loss rate for GPP buffer management policy.

**Figure 10:**
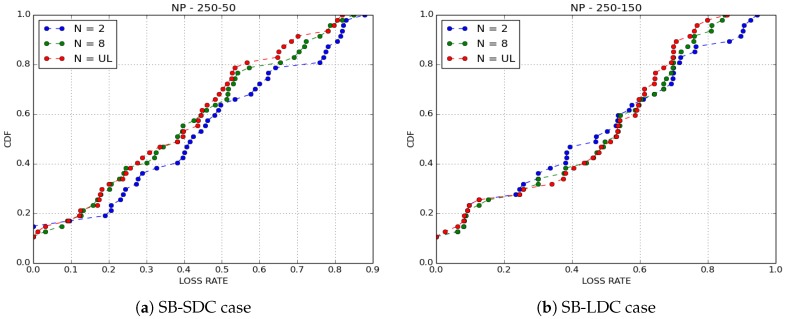

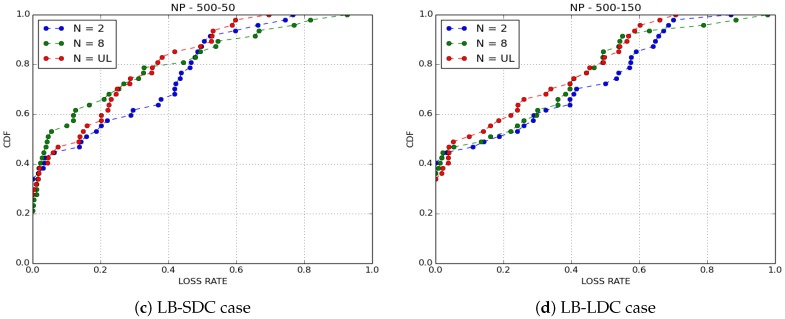
Loss rate for NP buffer management policy.

In Figure 9 and Figure 10, we notice that Epidemic routing protocol provides the best performance in terms of loss rate in all cases. Indeed, the redundancy and the robustness are maximized by diffusing a large number of copies in the network. Here, it is important to point out that GPP outperforms NP in all simulated scenarios, this is because of its protection to own production. Binary Spray and Wait has similar findings than Epidemic protocol whilst Two-Hop Relay protocol is the worst. This behaviour can have two explanations: one consists of the wrong choice of the neighbor node chosen as relay to forward the packet to a base station, another reason can be the need of more than two hops to send the redundant packet.

**Figure 11:**
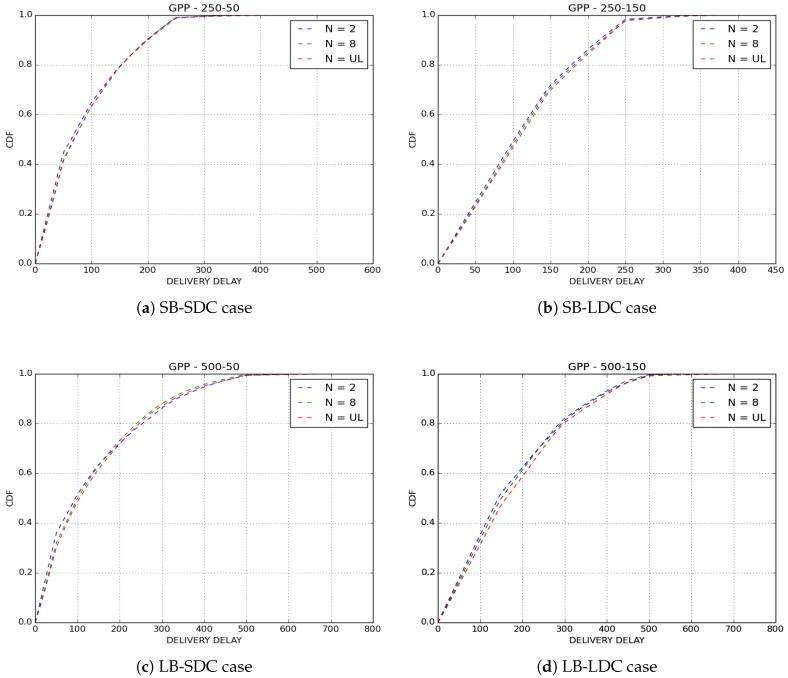
Delivery delay for GPP buffer management policy.

**Figure 12:**
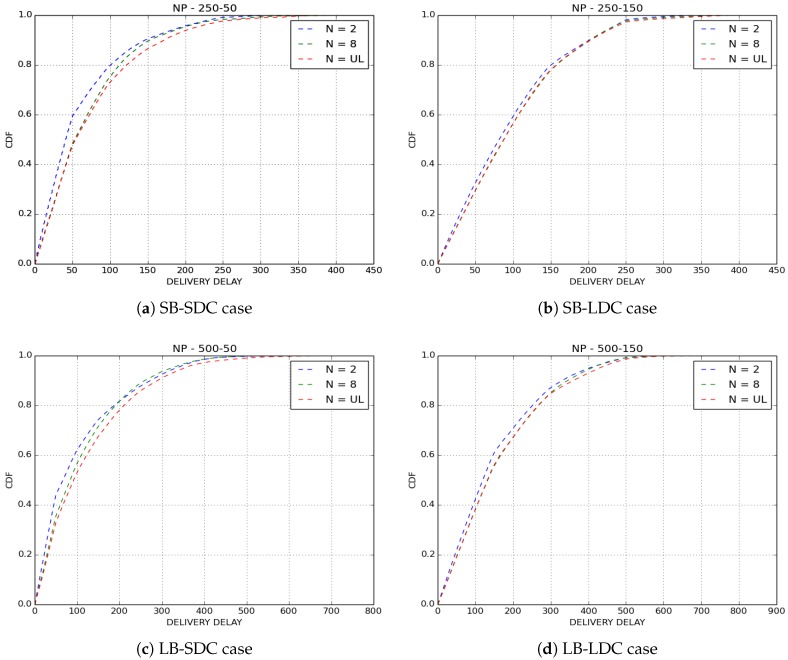
Delivery delay for NP buffer management policy.

Figure 11 and Figure 12 show the delivery delays of received packets for GPP and NP policies by varying the number of copies sprayed in the network. The first observation we make is that the three variants of IoB-DTN protocol offer similar performances. More precisely, Two-Hop Relay protocol provides the lower delivery delay, whereas Epidemic routing protocol is the worst. This is an expected result since Binary Spray and Wait and Epidemic protocol disseminate more packets in the network so they deliver older ones which degrades the overall delivery delay.

### 6.3. Impact of IoB-DTN by Varying the Transmission Power

**Figure 13:**
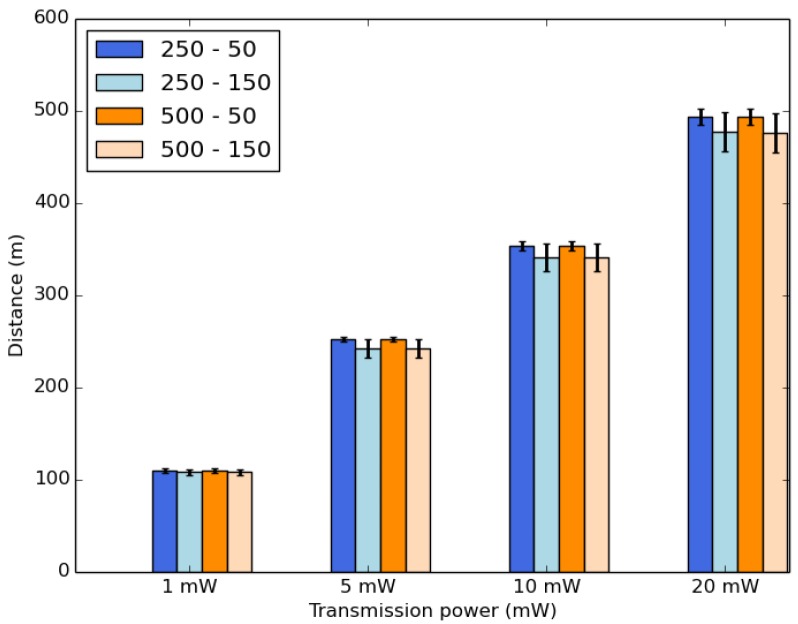
Average communication range.

Now, we evaluate IoB-DTN protocol by varying the transmission power of sensors. It is a significant parameter for IoB-DTN since it can influence on the communication range of sensors, as shown in Figure 13, thus influencing several metrics. In our previous simulated scenarios, the transmission power value was set to 10 mW which gives ∽350 m as communication range. Here, we appraise fours values of this parameter: 1 mW, 5 mW, 10 mW and 20 mW. We present the average results obtained for ten scenarios simulated with different paths of bikes in each scenario. In Figure 13, the highest value of the transmission range achieves the longest communication range.

**Figure 14:**
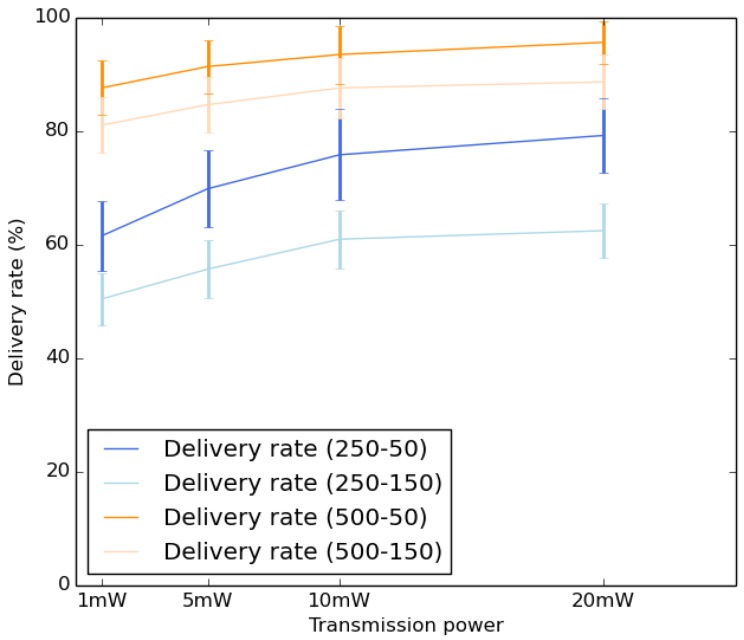
Average delivery rate.

Figure 14 shows the average delivery rate by varying the transmission power value. We notice that the delivery rate increases by raising the transmission power value. This behavior was expected, as, by increasing the communication range, each node will encounter more nodes and more base stations, hence allowing to obtain a higher delivery rate. It is also interesting to remark that the use of lower duty cycle improves the delivery rate.

**Figure 15:**
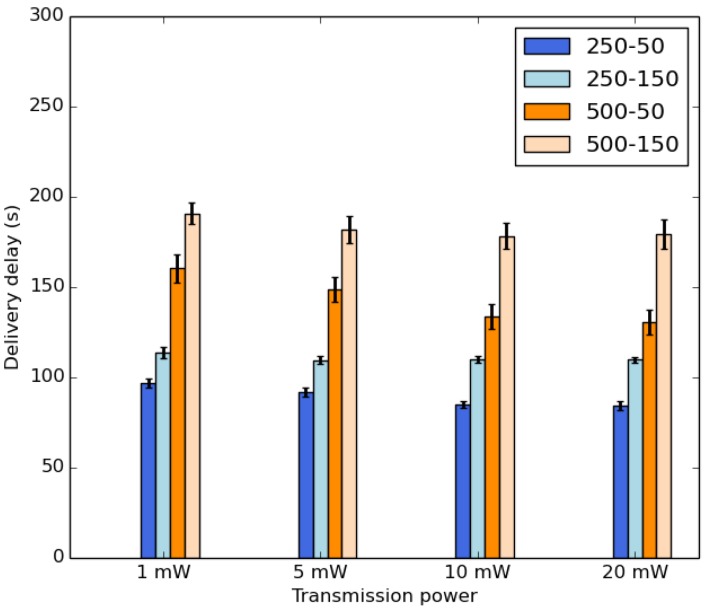
Average delivery delay.

The average delivery delay of received packets is depicted in Figure 15. It is important to point out that the transmission power does not impact the delivery delay comparing it to the buffer size and the duty cycle parameters. This indicates that the dynamics of the network impacts more the connectivity than the sending range. The increase of the buffer size as well as the duty cycle provide a higher delivery delay. In that case, more data packets are stored in the buffer for a longer time.

**Figure 16:**
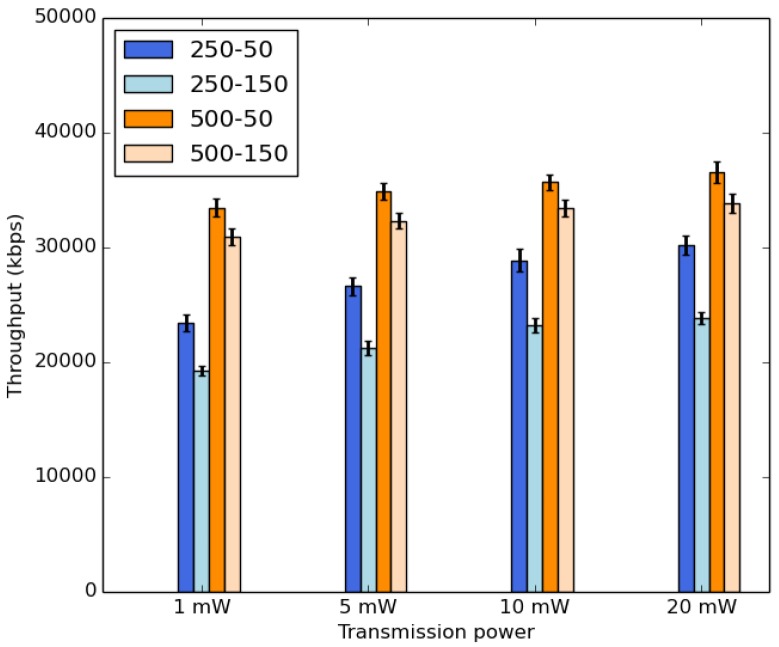
Average throughput.

Figure 16 shows the average throughput. Here, we note that the throughput is impacted by the transmission range. It increases slightly by raising the transmission power values. It is important to mention that the impact of the buffer size and the duty cycle is still significant since all packets stored in the buffer are forwarded at each duty cycle.

**Figure 17:**
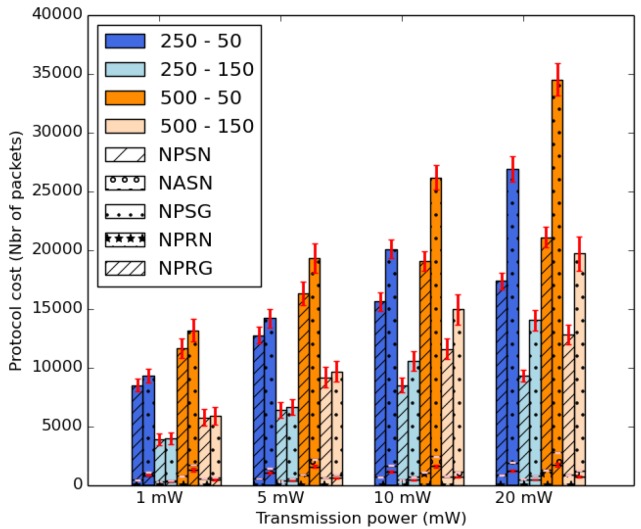
Average protocol cost.

Figure 17 shows the average protocol cost of IoB-DTN protocol by varying the transmission power values. It represents the average number of transmitted and received packets in all simulated scenarios. We consider all communications either bike-to-bike or bike-to-bike station. We can see two columns for each case: the first column depicts all transmitted packets and the second represents all received packets. On the one hand, the first column has three fields: *NPSN* (number of packets sent to nodes), *NASN* (number of acknowledgments sent to nodes) and *NPSG* (number of packets sent to gateways). On the other hand, the second column has two fields: *NPRN* (number of packets and acknowledgments received by nodes) and *NPRG* (number of packets received by gateways). Figure 17 clearly illustrates the impact of the increase of the transmission power value on the average number of forwarded and received data packets in the network. This is an evident consequence since the transmission range increases by raising the transmission power of sensors which will encounter more nodes and more base stations. Hence, this allows more communications with other nodes and gateways. It is also interesting to notice that the use of a high duty cycle offers a small duty cycle. Indeed, in that case, the data packets spend more time to be stored in the buffers, thus allowing to have fewer communications.

According to the results obtained in this section, we can argue that IoB-DTN protocol with a high transmission power and a small duty cycle can provide a better delivery rate, delivery delay and throughput. It has a high energy performance. The value of the transmission power of sensors is chosen depending on the needs of the designers of the network. In next sections, we consider 10 mW as transmission power since it offers the compromise among all evaluated metrics.

## 7. Performance Evaluation of IoB-DTN and IoB Long-Range

**Figure 18:**
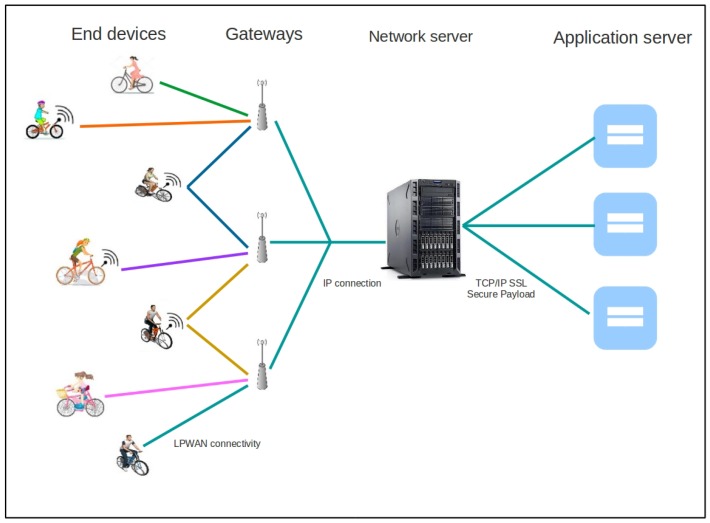
Illustration of IoB Long-Range.

In this section, we are interested in comparing the performance of IoB-DTN protocol and a low-power long-range technology. IoB-DTN protocol is a multi-hop protocol since there are bike-to-bike and bike-to-bike station communications. In a long range technology, only bike-to-bike station communication is allowed, as shown in Figure 18. For that, we consider IoB-DTN protocol with a radio propagation that provides us ∽1 km as communication range, it is denoted IoB-Long Range (IoB-LR). By following IoB-LR, each node generates periodically data packets and stores them in its buffer. When the duty cycle is over and when the node lies in the area of a base station, it sends all data stored in its buffer.

Here, we assume that IoB-LR represents a low-power long-range technology, LoRa type. More precisely, we suppose that IoB-LR follows the model of LoRa Semtech SX1272 chipset [48]. As for IoB-DTN protocol, we consider, for our theoretical results, the parameters offered by the Qualcomm AR6004 802.11p chipset [49]. The parameters used for both considered models are depicted in Table 3.

**Figure 19:**
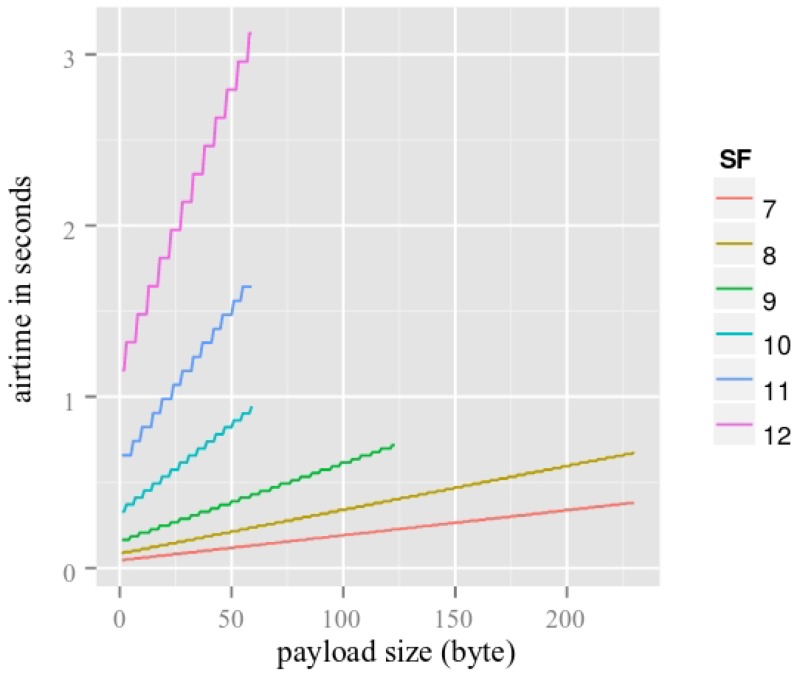
Airtime for different SF and payloads [50].

It is important to notice that the packet duration for the long range, LoRa type, varies according to the four considered cases. As an example, considering the SB-SDC scenario: 250 as buffer size and 50 s as duty cycle, the 250 packets stored in the buffer have to be sent during the 50 s time frame of the duty cycle. Therefore, each packet needs to have a maximum airtime of 0.2 s. It is also interesting to point out that the packet size varies for each case for IoB-LR. To better understand this, we refer the reader to Figure 19 which shows the airtime in seconds (time to forward a packet) for different spreading factors SF (length of the code sent) and payload size given by an operational LoRaWAN, namely The Things Network [50]. In this work, the bandwidth used is 125 KHz and the coding rate (forward error correction) is $\frac{4}{5}$. The data in Figure 19 are used to determine the packet size for each case for IoB-LR. The values of the payload sizes obtained for different spreading factors for each case are presented in Table 4.

As mentioned above, the spreading factor represents the number of chirps used to encode a bit and it varies from *SF7* to *SF12*. The higher is the chirp rate, the better is the reconstitution of the received signal, while it has a significant delay to forward a bit. We consider payload sizes with the spreading factor SF7 for IoB-LR in all our scenarios since it provides the highest values.

**Figure 20:**
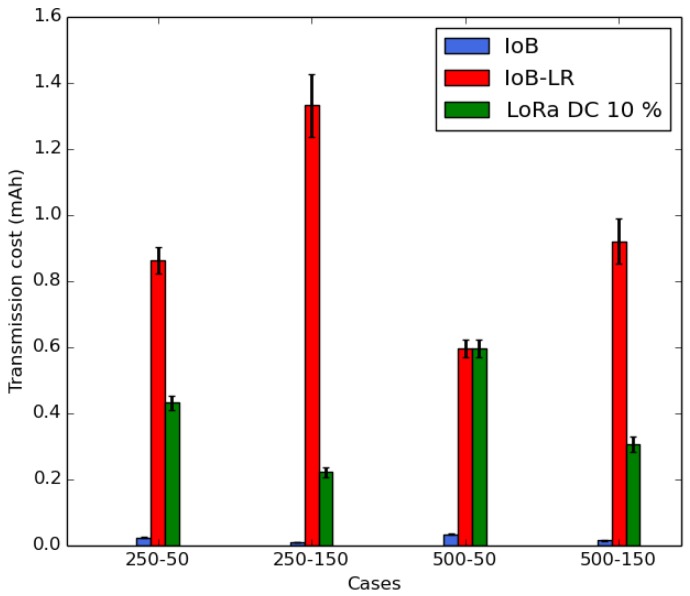
Average transmission cost per bike.

In this section, we compare the energy consumption and the throughput of IoB-DTN and IoB-LR. To evaluate the energy consumption, we assess the average transmission cost per bike and the average consumption background per bike. The average transmission cost per bike is shown in Figure 20. It is measured in mAh and calculated as follows:

(1) $$TC=\left[NPS*Tx*DP\right]+\left[NAR*\left(Tx+Rx\right)*DA\right]+\underset{\;\mathrm{for}\;\mathrm{IoB}}{\underset{︸}{\left[NPR*Rx*DP\right]}}$$

where *TC* represents the transmission cost expressed in mAh; *NPS* is the number of sent packets; *Tx* and *Rx* correspond to the transmit and receive consumption, respectively, measured in mA; *DP* and *DA* represent the packet duration and the acknowledgment duration calculated in seconds, respectively; *NAR* is the number of received acknowledgments; and *NPR* is the number of received packets from nodes.

In Figure 20, we notice that IoB-LR gives the highest average transmission cost per bike. This is because nodes forward data packets to only base stations. IoB protocol offers the smallest average sending cost per bike by dint of the multi-hop communications that decrease the forwarding cost per bike in the network. To respect the duty cycle (real period in which a resource is active) of radio devices regulated in Europe by Section 7.2.3 of the ETSI EN300.220 standard, we consider the maximum theoretical duty cycle allowed for the long range type LoRa which is 10% using the sub-bands (869.4–869.65 MHz) [51]. Thereby, we present, in Figure 20, the average forwarding cost per bike for LoRa technology by respecting the maximum theoretical duty cycle of 10%. From the simulated scenarios, the LB-SDC case, with 500 as buffer size and 50 s as duty cycle, corresponds to the real value of the maximum duty cycle allowed by LoRa technology. In other words, to fill $\frac{1}{10}$ of 500 slots in 50 s, the duty cycle is then 10%. By following the same behavior for other cases, the duty cycles obtained are: 20% for SB-SDC, 60% for SB-LDC and 30% for LB-LDC. As final note for the transmission cost, it is important to mention that IoB gives the best results in terms of the average transmission cost per bike, thanks to the bike to bike communication, comparing to IoB-LR and LoRa technology by respecting the maximum allowed duty cycle.

**Figure 21:**
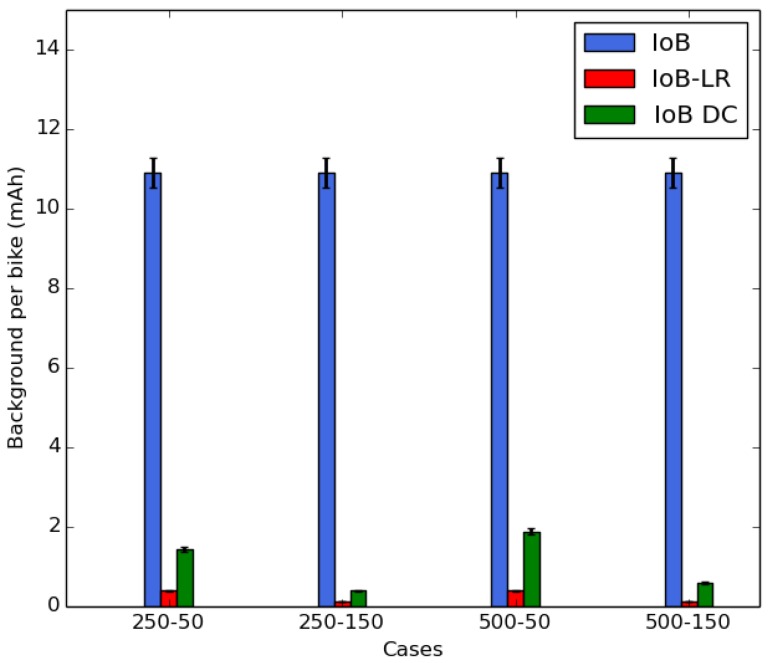
Average consumption background per bike.

The average consumption background per bike is depicted in Figure 21. It corresponds to the overall consumption by bike, measured in mAh, and is calculated as follows:

For IoB:

(2) BC=∑(Ta−Td)∗Rx

For IoB-LR:

(3) $$BC=\frac{Rx*BWT}{DC}*\sum \left(Ta-Td\right)$$

where *BC* represents the average background consumption; *Ta* and *Td* are the arrival and the departure time of the bicycle, respectively, defined in seconds; and *DC* corresponds to the duty cycle expressed in seconds. After this period, each node forwards all data stored in its buffer. It takes as value 50 s or 150 s according to the simulated scenario. *BWT* represents the beacon waiting period measured in seconds and it is fixed to 10 s in all our measures.

In Figure 21, we notice that IoB-DTN protocol has the highest average background consumption per bicycle. This is because the nodes require to be always in listening mode for beacons to relay data packets in the network. For IoB-LR, the nodes enter in sleep mode and they wake up few moments before starting the data packets sending. We also present, in Figure 21, an optimal average background consumption per bicycle for IoB and it is denoted IoB-DC. In IoB-DC, the nodes having a full buffer enter in sleep mode and they wake up few seconds before data packets forwarding. We note that IoB-LR has better background consumption per bike than IoB and IoB-DC. In Figure 20 and Figure 21, IoB-LR, based on the long range technology, clearly provides lower energy consumption than the multi-hop IoB-DTN protocol.

**Figure 22:**
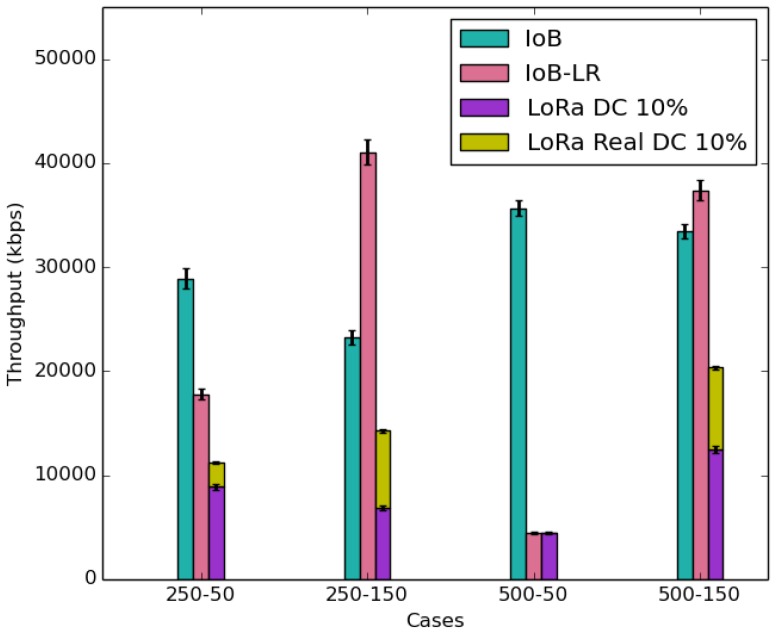
Average throughput.

Figure 22 shows the average throughput expressed in *kbps*. It is interesting to notice that IoB has better throughput when the duty cycle is small, whereas IoB-LR offers a higher throughput when the duty cycle is high. In fact, these results are related to the packet sizes transmitted in each simulated scenario. We remember that the packet sizes, with SF7, chosen for IoB-LR with SB-LDC and LB-LDC cases are higher than the packet size of IoB.

**Figure 23:**
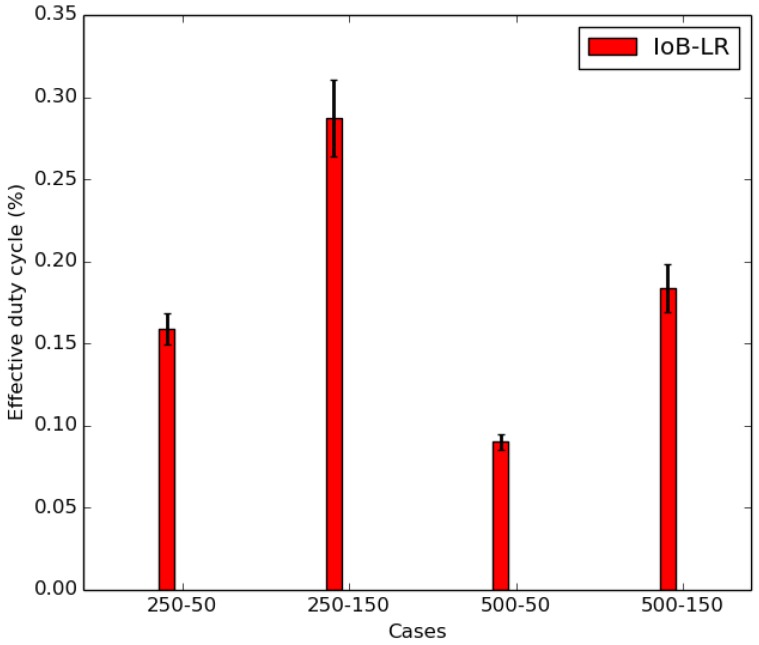
Average effective duty cycle.

In Figure 22, we also present the average throughput for IoB-LR with the respect of the maximum theoretical duty cycle allowed by LoRa technology. We observe that IoB offers the best throughput than IoB-LR that respects the theoretical duty cycle in all simulated scenarios. Figure 22 shows the average throughput for IoB-LR when respecting the effective duty cycle. It corresponds to the real duty cycle of the bicycles in each scenario. It is shown in Figure 23 and is computed as follows:

(4) $$EDC=\frac{NPS*Rt}{\sum (Ta-Td)}$$

where *EDC* represents the effective duty cycle; *NPS* is the number of sent packets; and *RT* corresponds to the airtime defined in seconds. We take for example the case for SB-SDC, the duty cycle should be 20% as mentioned before. On the other hand, its real duty cycle is ∽16%. Hence, it is important to point out that IoB-DTN protocol provides better throughput than IoB-LR and IoB-LR respecting the theoretical and the effective duty cycle.

In the next section, we propose a solution for IoB-DTN protocol in order to improve its performance on energy consumption.

## 8. Performance Improvement of IoB-DTN Based on Data Aggregation Mechanism

In this section, we investigate an efficient multi-hop IoB-DTN protocol based on data aggregation approach being applied to mobile network IoT devices running a data collection application in order to enhance its performances. The idea is to combine data packets of different nodes into a single packet. This mechanism is performed during the generation and the reception of a new data packet, which are depicted in Algorithms 2 and 3, respectively. We propose three variants of IoB-DTN protocol:

- *Spatial aggregation (IoB-SA)*: Data packets are aggregated if they were generated in the same area whose its range is defined in meters.
- *Temporal aggregation (IoB-TA)*: Data packets are aggregated if they were generated less than a period defined in seconds earlier or later than the reference packet.
- *Spatiotemporal aggregation (IoB-STA)*: Data packets are aggregated if they satisfy the two preceding conditions.

**Algorithm 2***Generation phase*: IoB-DTN based on data aggregation.
1:At each sensor reading period2:Generate a packet *p* with the readings3:**if** (Δ (*p*, $p^{\prime }$) < (Δ area || Δ period || (Δ area + Δ period) ) ) **then**4: Aggregate *p* with the packet $p^{\prime }$5:
**else**
6: **if** Buffer management provides a slot **then**7:  Store $p\cup N^{0}$ in the buffer [$N^{0}$ copies of *p* are stored]8: **end if**9:
**end if**



**Algorithm 3***Reception phase*: IoB-DTN based on data aggregation.
1:On reception of packet p∪N∪L (list of neighbors)2:pos← self position in *L*3:
$N^{\prime }\leftarrow \frac{N}{2^{pos+1}}$
4:**if** (Δ (*p*, $p^{\prime }$) < (Δ area || Δ period || (Δ area + Δ period) ) ) **then**5: Aggregate *p* with the packet $p^{\prime }$6:
**else**
7: **if** Buffer management provides a slot **and** $N^{\prime }\geq 1$ **then**8:  Store $p\cup N^{\prime }$9:  Send ACK for receiving $N^{\prime }$ copies of *p*10: **else**11:  Packet is rejected, no ACK is sent12: **end if**13:
**end if**



Based on the previous section, we suppose here that the data packet has size of 20 bytes that could be forwarded by a low-power long-range technology as LoRa/LoRaWAN. Considering the 802.11p packet size having as a value 160 bytes, we can identify two approaches:

- *Without size reduction aggregation*: Eeach node can combine up to seven data packets into the same packet coming from various neighbors nodes without data processing.
- *With size reduction aggregation*: Each node receives more than seven data packets to be combined into the same packet, and compresses their readings. In other words, each node performs the basic aggregation functions to process data to limit the number of data packets aggregated into the same packet. To accomplish that, simple functions are used such as the Average, SUM, COUNT, MAX, MIN, etc. The use of this operation depends on the type of data collection application. This paper does not consider the aggregation function since our protocols are independent of it.

Next, we gives a performance evaluation of IoB-DTN protocol based on data aggregation approach. More precisely, we compare the three variants of IoB mentioned before. We assess four metrics: delivery rate, delivery delay, throughput and energy consumption. We remember that we present the average results for ten scenarios simulated by varying the paths of bicycles in each scenario.

### 8.1. Spatial Aggregation

The aggregation area is the space where the sensed values by various sensor nodes are generated. We evaluated three values of the aggregation distances: 20 m, 50 m and 100 m.

**Figure 24:**
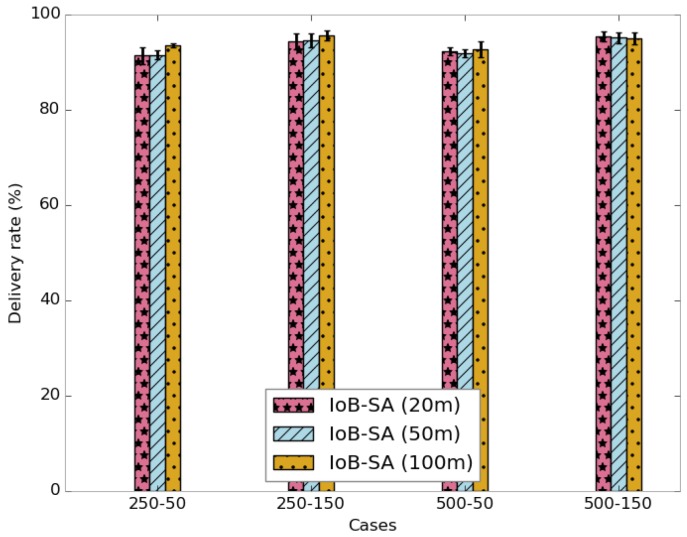
Average delivery rate for IoB-SA.

**Figure 25:**
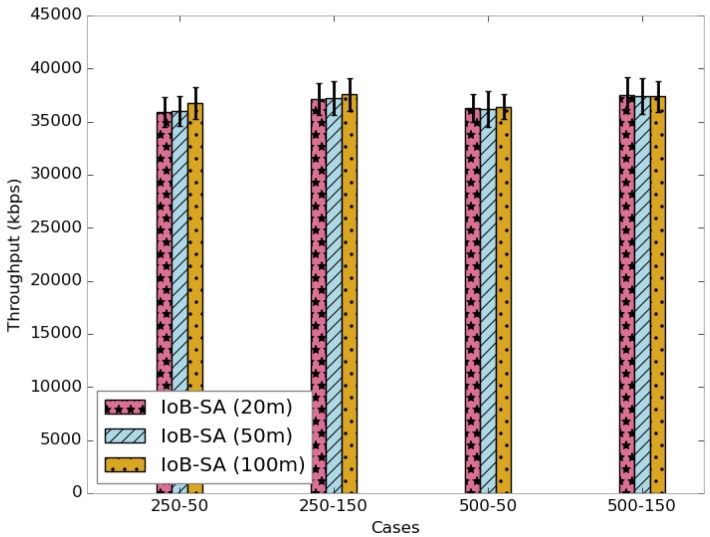
Average throughput for IoB-SA.

Figure 24 shows the average delivery rate for spatial aggregation. We observe that the increase of the aggregation area does not impact the delivery rate. More precisely, the forwarding rate raises slightly by increasing the aggregation distance. Moreover, the throughput is not impacted by the rise of the aggregation distance, as illustrated in Figure 25. This indicates that the dynamics in the network is more impacted than the aggregation area.

**Figure 26:**
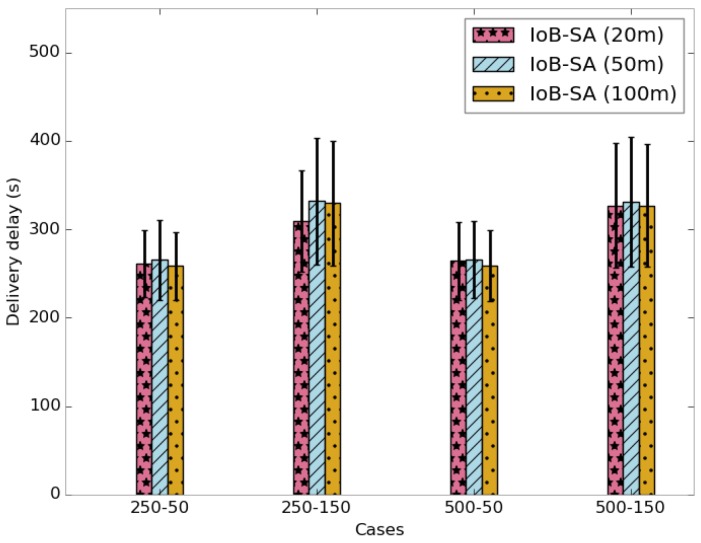
Average delivery delay for IoB-SA.

Figure 26 depicts the average delivery delays of received data packets. Again, we notice that the delivery delays of the three parameters are almost the same. It is important to point out that using a small duty cycle is better since the packets are forwarded faster.

**Figure 27:**
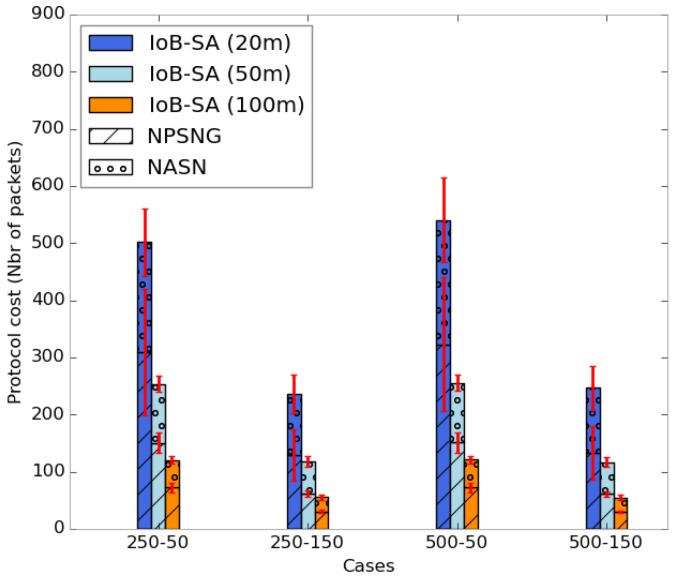
Average protocol cost for IoB-SA.

To evaluate the energy consumption of IoB-DTN protocol with spatial aggregation, we appraise the protocol cost and the number of packets aggregated per packet. Figure 27 shows the average protocol cost of IoB-SA variant. It is calculated as the number of data packets forwarded in the network and is depicted in Figure 27. We can see two fields in each column: *NPSNG* (number of packets sent to nodes and gateways) and *NASN* (number of acknowledgments sent to nodes). It is clear that the increase of the aggregation area reduces the overall protocol cost in the network. This is an obvious consequence since more data packets are aggregated into same packets, thus achieving better communication cost. In addition, the use of a large value of duty cycle reduces the protocol cost since data packets will be stored for a longer period in the buffers.

**Figure 28:**
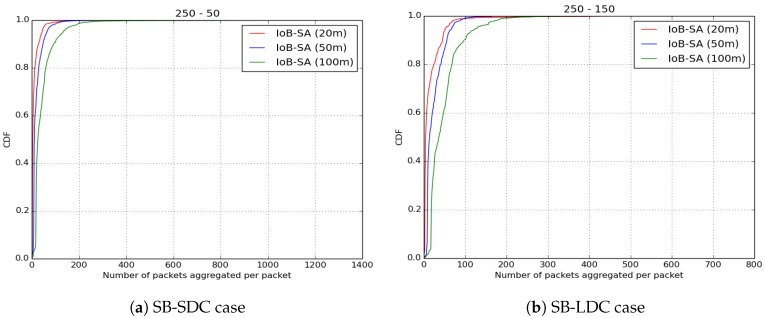

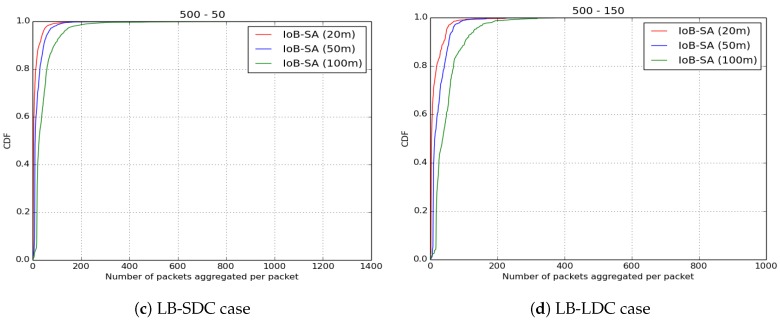
Average number of aggregated packets per packet for IoB-SA.

Moreover, to assess the communication cost as well as investigate the energy consumption of IoB-SA, we also evaluate the number of aggregated packets into the same packet for the three parameters, as illustrated in Figure 28. As cited above, when a node receives more than seven data packets to be aggregated into the same packet, it applies the aggregation functions. This can influence on the energy consumption of sensor nodes, therefore the overall network lifetime. It is important to notice that using a small aggregation distance (20 m) leads to aggregating fewer data packets into the same packet since the source node meets fewer neighbors nodes.

### 8.2. Temporal Aggregation

The aggregation period is the duration, defined in seconds, where the sensed values by various sensor nodes are generated earlier or later than the reference packet. We assessed three values of the aggregation period: 2 s, 5 s and 10 s.

**Figure 29:**
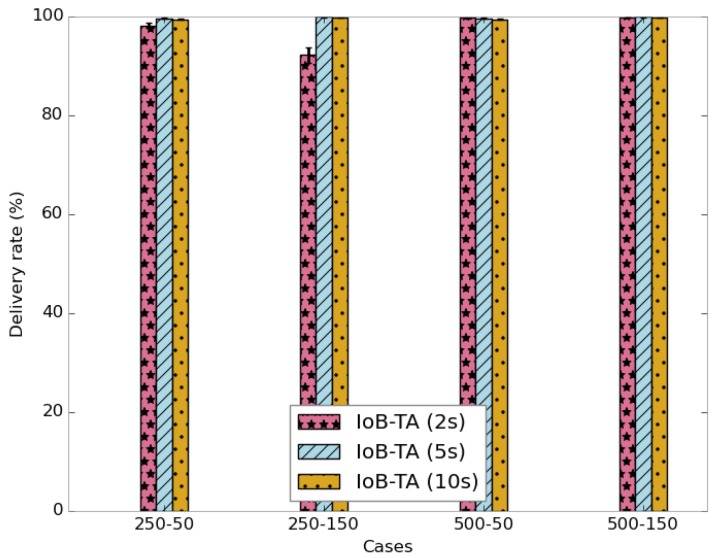
Average delivery rate for IoB-TA.

**Figure 30:**
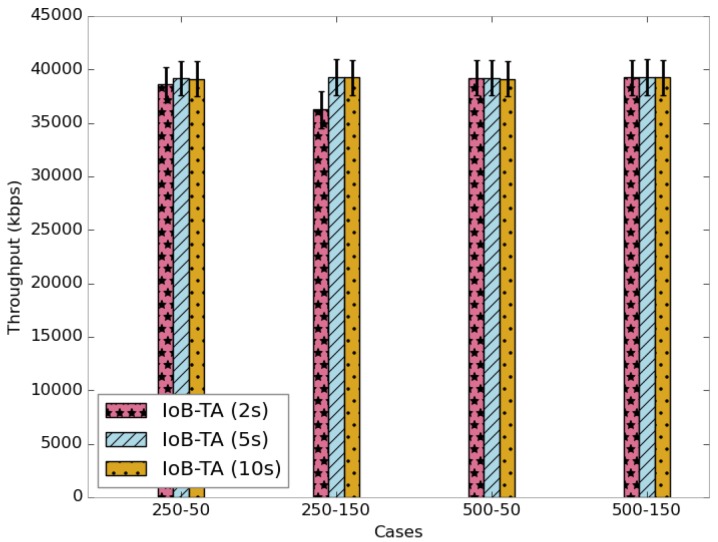
Average throughput for IoB-TA.

The average delivery rate of IoB-TA variant is shown in Figure 29. The first observation we make is the achievement of 100% as transmitting rate by using a large buffer. The use of a small buffer size and increasing the aggregation period slightly increase the delivery rate. Here, it is worth pointing out that the delivery delay results obtained using temporal aggregation are better than using spatial aggregation. In the same context, these outcomes influence the throughput, as depicted in Figure 30.

**Figure 31:**
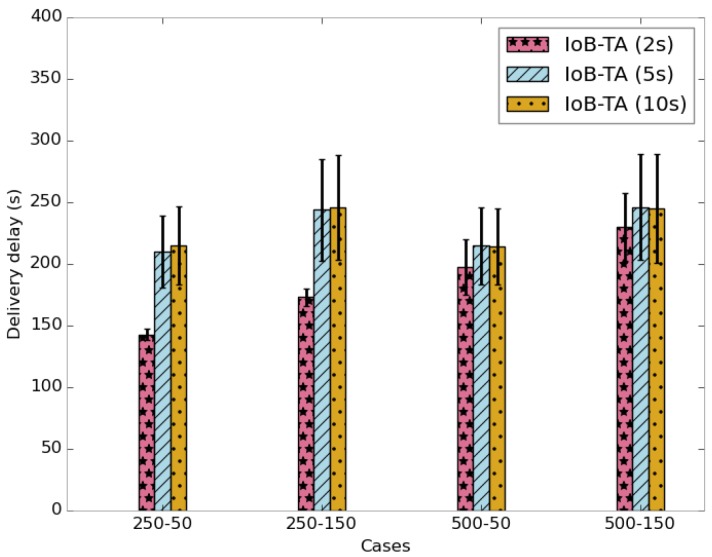
Average delivery delay for IoB-TA.

The average delivery delays of received packets is shown in Figure 31. We note that the delivery delays increases progressively when rising the aggregation period. As expected, the use of a small duty cycle offers lower delays since data packets are delivered faster.

**Figure 32:**
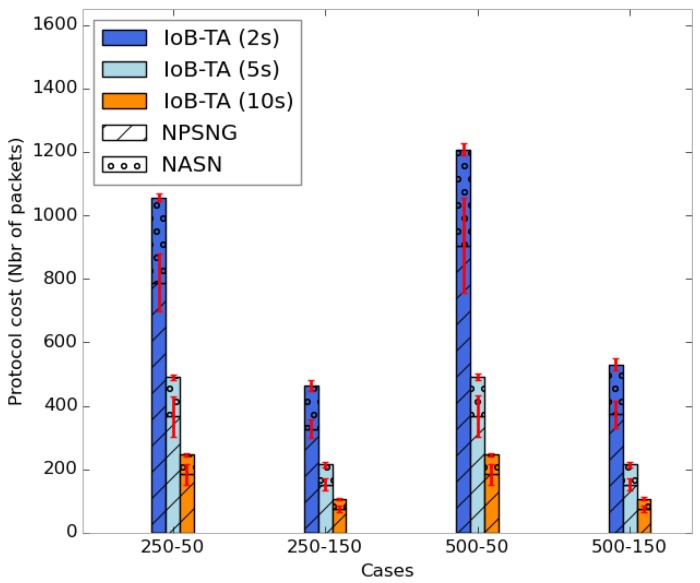
Average protocol cost for IoB-TA.

As for the energy consumption of IoB with temporal aggregation, we also evaluate the two parameters cited before. The average protocol cost is illustrated in Figure 32. We remark that the number of transmitted packets in the network decreases by increasing the aggregation period. As for spatial aggregation, the rise of the aggregation parameter leads to having more packets aggregated which reduces the overall protocol cost in the network. It is important to note that the communication cost for IoB with temporal aggregation is twice that of IoB with spatial aggregation.

**Figure 33:**
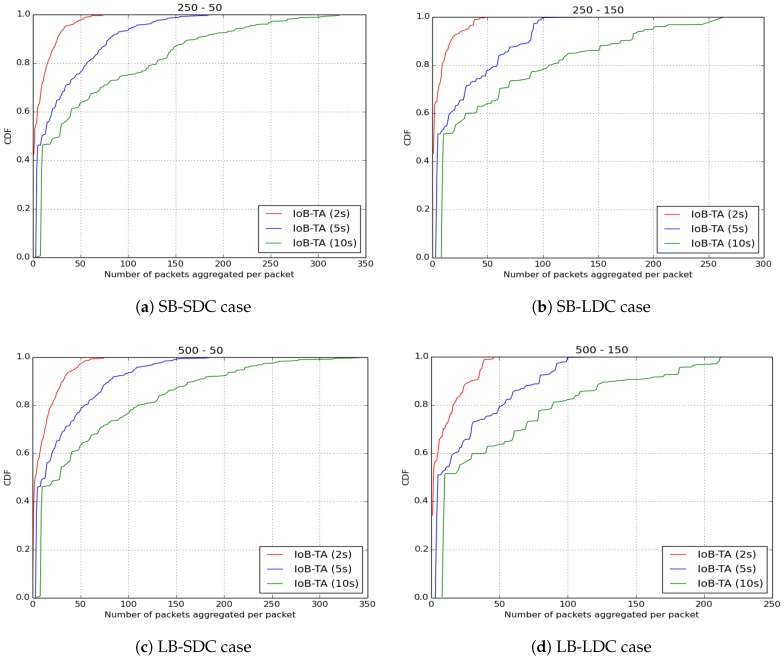
Average number of aggregated packets per packet for IoB-TA.

The number of aggregated packets into the same packet for IoB with temporal aggregation is depicted in Figure 33. As for spatial aggregation, the use of the smallest period value (2 s) gives better performance since it leads to aggregating fewer data packets into a packet.

### 8.3. Spatiotemporal Aggregation

In spatiotemporal aggregation, the data packets are aggregated if they satisfy the two conditions of the aggregation area and the aggregation period mentioned above. We evaluated four values of IoB-STA variant: 20 m–2 s, 100 m–2 s, 20 m–5 s and 100 m–5 s.

**Figure 34:**
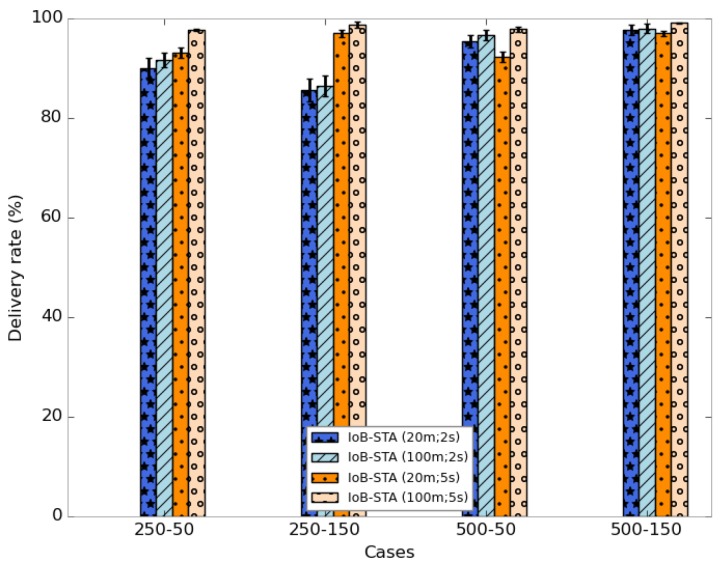
Average delivery rate for IoB-STA.

**Figure 35:**
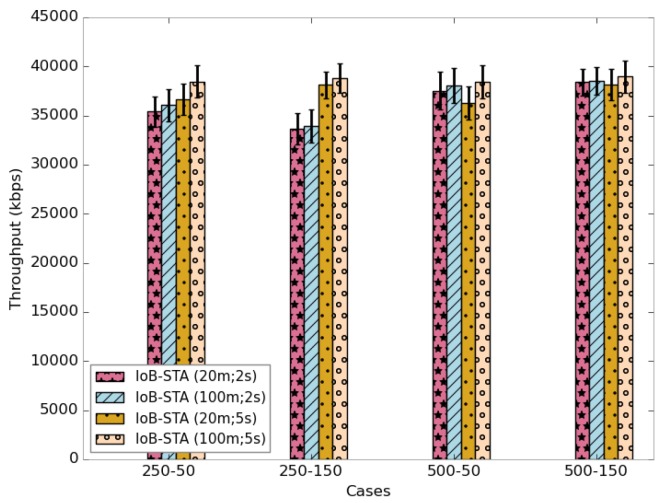
Average throughput for IoB-STA.

Figure 34 shows the average delivery rate of IoB with spatiotemporal aggregation. As expected, we observe that using higher values of both aggregation parameters offers better performance. In addition, we can notice that using a high value of the aggregation period gives better results than lower one. This indicates the efficiency of temporal aggregation approach. This result impacts the throughput, as presented in Figure 35.

**Figure 36:**
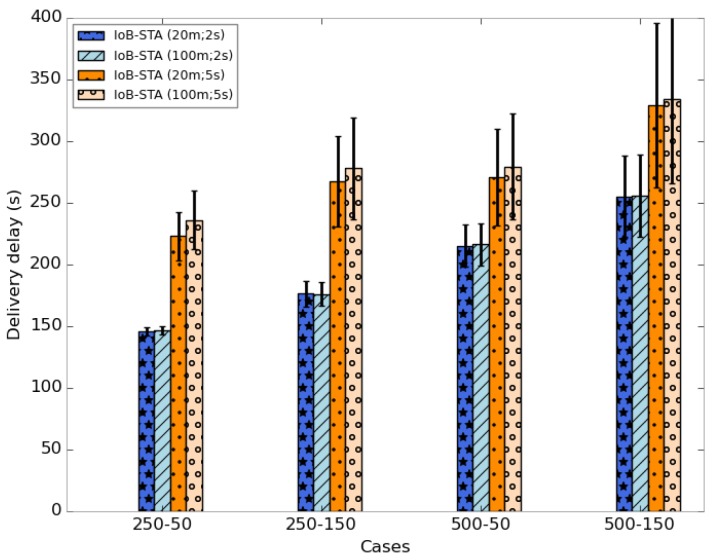
Average delivery delay for IoB-STA.

The average delivery delays are shown in Figure 36. It is clear that the use of small aggregation parameters offer lower delays. It is important to note that using a small buffer size provides the best outcomes in terms of delivery delay.

**Figure 37:**
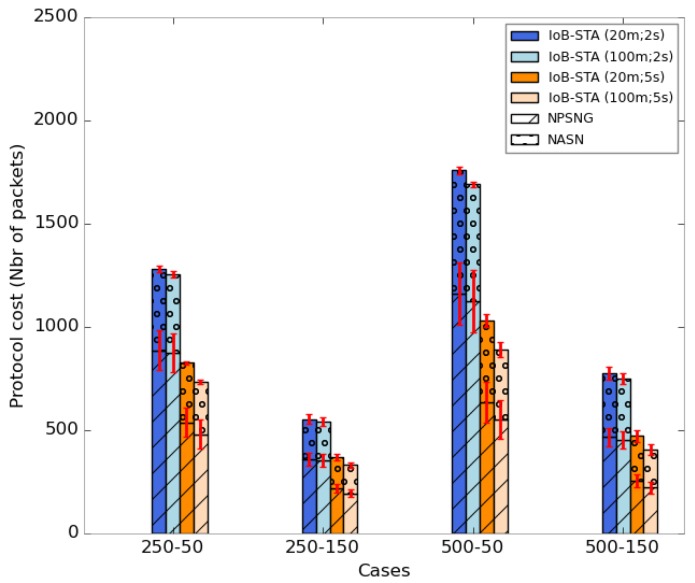
Average protocol cost for IoB-STA.

Figure 37 depicts the average protocol cost of IoB-STA. We notice that more the aggregation parameters values are higher more the protocol cost is better. This is an expected consequence since more data packets will be aggregated into same packets which decreases the communication cost.

**Figure 38:**
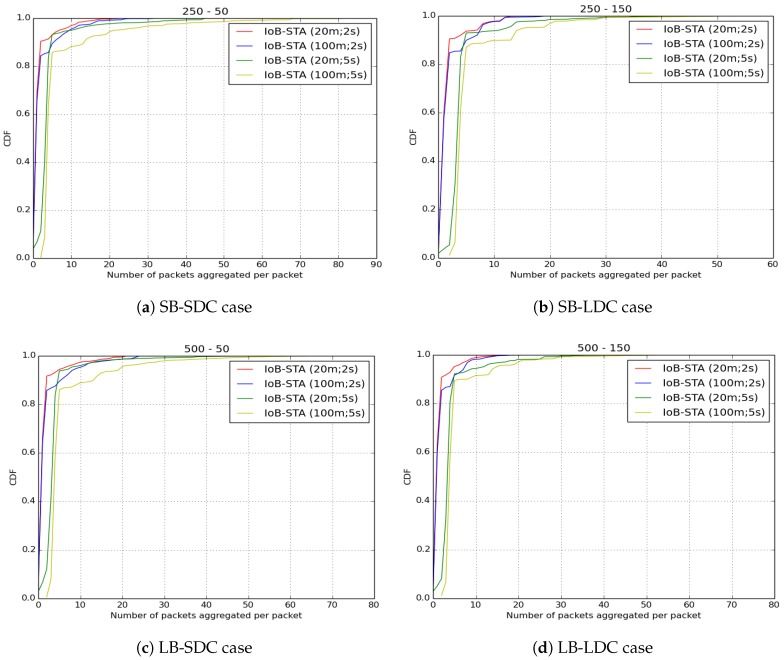
Average number of aggregated packets per packet for IoB-STA.

As for the number of aggregated packets into the same packet, as shown in Figure 38, we note that using the smallest aggregation values (20 m–2 s) offer the best results since they lead to aggregating fewer packets which improves the energy consumption of sensor nodes.

### 8.4. Performance Comparison of Six Variants of IoB

Here, we compare six variants of IoB-DTN protocol: the multi-hop IoB without data aggregation, IoB with one hop, IoB-Long range, IoB based on spatial aggregation, IoB based on temporal aggregation and IoB based on spatiotemporal aggregation. IoB with one hop behaves similar to the multi-hop IoB without aggregation except only bike-to-bike station communication is applied. The difference between IoB with one hop and IoB-LR is the communication range value. For the first variant, we keep the same communication range (∽350) as the multi-hop IoB without aggregation, whereas we consider, as discussed before, a radio propagation that provides ∽1 km as communication range for IoB-LR.

From the results obtained above, spatial aggregation with 20 m as aggregation area, temporal aggregation with 2 s as aggregation period and spatiotemporal with 20 m and 2 s as aggregation parameters offer the best outcomes in all simulated scenarios. Thus, we give a comparison of these variants with these aggregation values.

**Figure 39:**
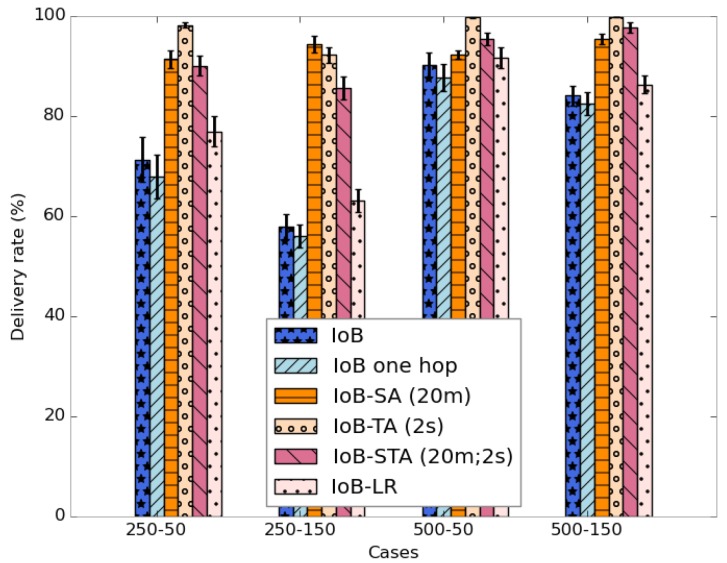
Average delivery rate comparison.

Figure 39 shows the average delivery rate of the six variants of IoB-DTN protocol. The first observation we make is that the three variants of IoB based on data aggregation mechanism provide better delivery rate. Indeed, combining data packets into a single packet increases the probability to reach destinations. The fact that the delivery rate is higher by using a large buffer size indicates that more data packets are stored in buffers and forwarded later to base stations. This shows that the aggregation mechanism used provides robustness. More precisely, IoB based on temporal aggregation offers better result than other variants and it achieves 100% when the buffer size is large. It is also interesting to note that spatial aggregation gives better performance in terms of forwarding rate than spatiotemporal aggregation when the buffer size is small and inversely when increasing the buffer size.

**Figure 40:**
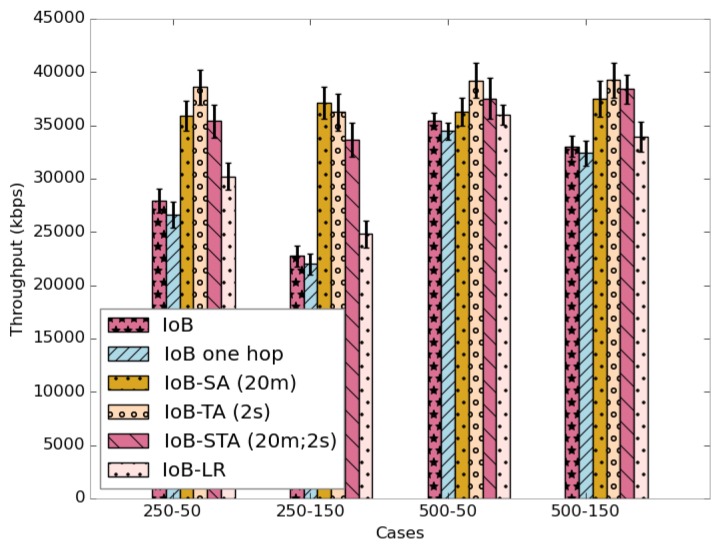
Average throughput comparison.

As for the throughput, as shown in Figure 40, the three variants of IoB based on data aggregation provide best performance.

**Figure 41:**
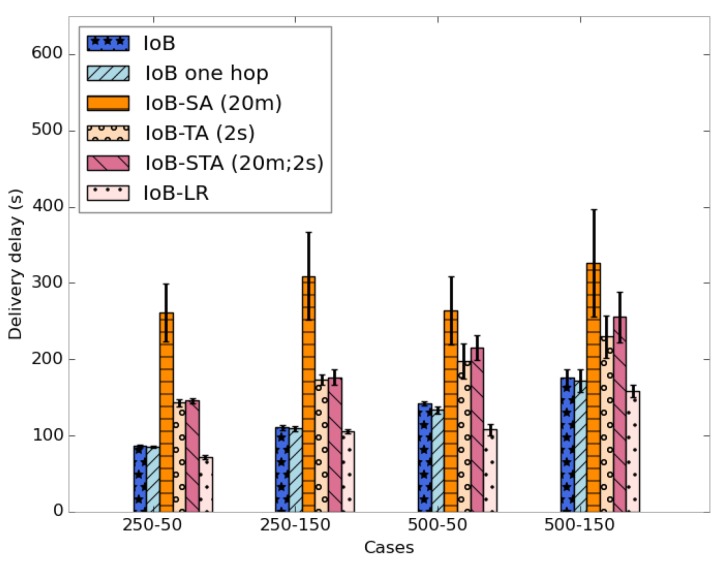
Average delivery delay comparison.

The average delivery delays are presented in Figure 41. Clearly, we notice that the three variants have the highest delays. The fact that data packets are combined generates a significant delay, which is an expected result. This may have as an explanation that data packets could be aggregated into packets in the buffers of the nodes having a lower probability to quickly meet base stations. More specifically, IoB based on spatial aggregation gives the worst delays.

**Figure 42:**
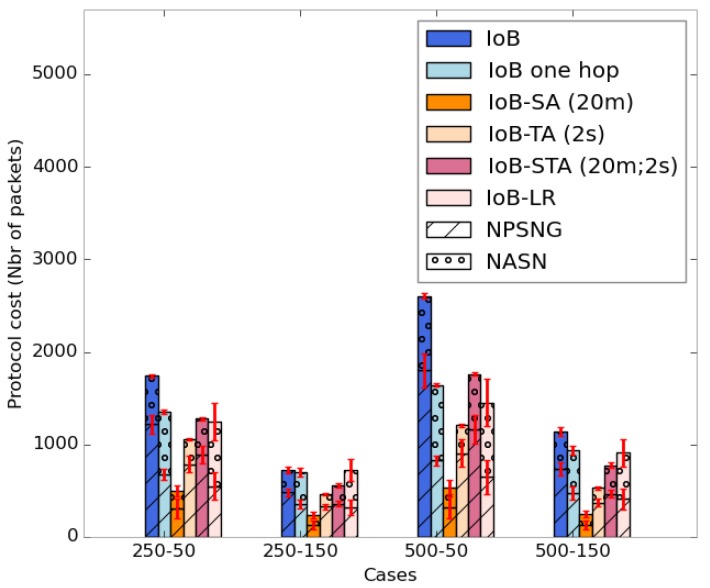
Average protocol cost comparison.

Figure 42 shows the average protocol cost of the six variants of IoB-DTN protocol. It is important to mention that applying data aggregation mechanism on IoB protocol reduces the communication cost in the network compared to other variants. More precisely, IoB with spatial aggregation offers the lower protocol cost.

**Figure 43:**
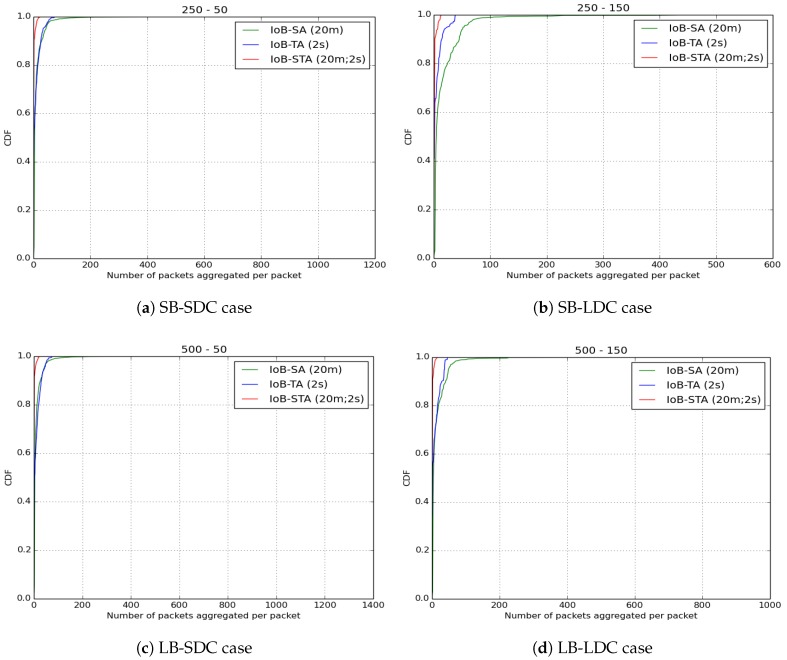
Average number of aggregated packets per packet for IoB-STA.

As for the number of aggregated packets into a single packet, which is illustrated in Figure 43, we compare the three variants of IoB based on data aggregation with the parameters mentioned at the beginning of this part. We remark that IoB with spatiotemporal aggregation is better than the other variants. It is also worth noting that IoB with temporal aggregation provides fewer packets aggregated per packet than IoB with spatial aggregation.

### 8.5. Discussion Results

**Figure 44:**
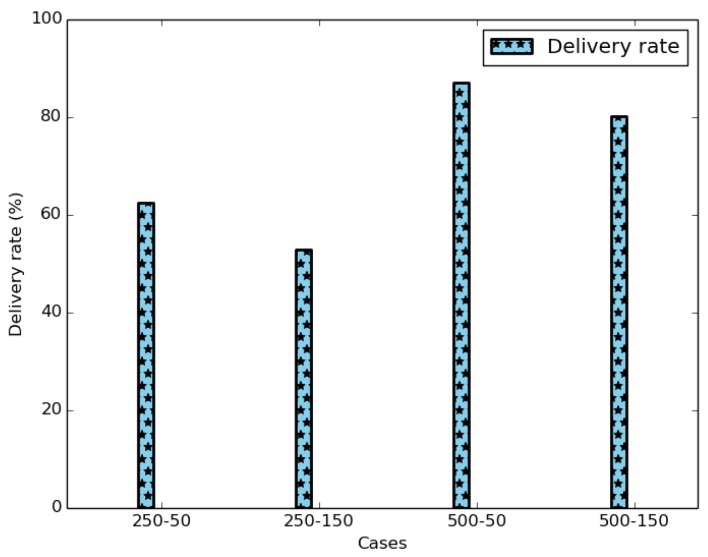
Loss rate.

**Figure 45:**
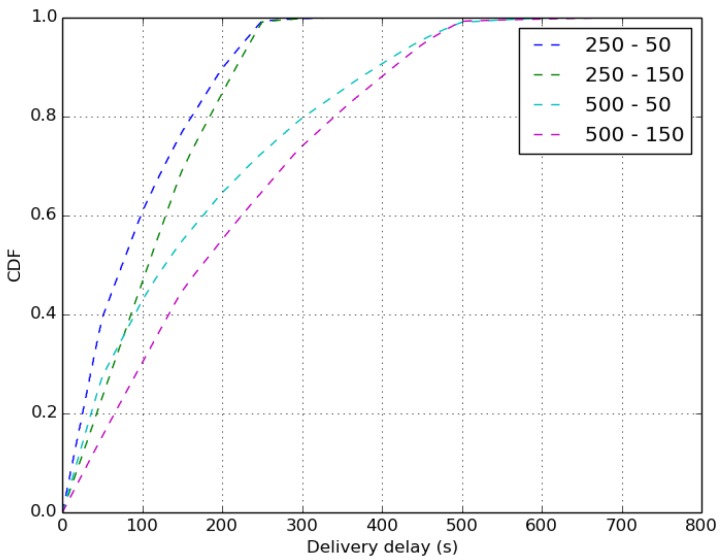
Delivery delay.

We recall that without aggregation the compromise between the delivery ratio and the delivery delay metrics is settled by the buffer size, as shown in Figure 44 and Figure 45. In these figures, we observe that the buffer size impacts the delivery rate and the delivery delay of received packets more than the duty cycle. More precisely, using small buffer sizes improves the delivery delay, as shown in Figure 45. In such case, the buffer will be full very fast and oldest packets are always dropped thanks to the GPP buffer management policy that gives priority to the self-generated packets. Using large buffer sizes enhances the delivery ratio as depicted in Figure 44. This is because there is more space to store generated and received packets, resulting in a higher transmission rate.

Based on the previous results cited in this section, we can argue that IoB-DTN protocol applying data aggregation approach improves the compromise between several metrics. It increases the delivery rate and the throughput as well as it saves the communication cost, and thus the network capacity. However, it does not impact much the delivery delay. It is interesting to point out that using IoB with aggregation provides less sensitivity to the buffer size regarding to all evaluated metrics.

**Figure 46:**
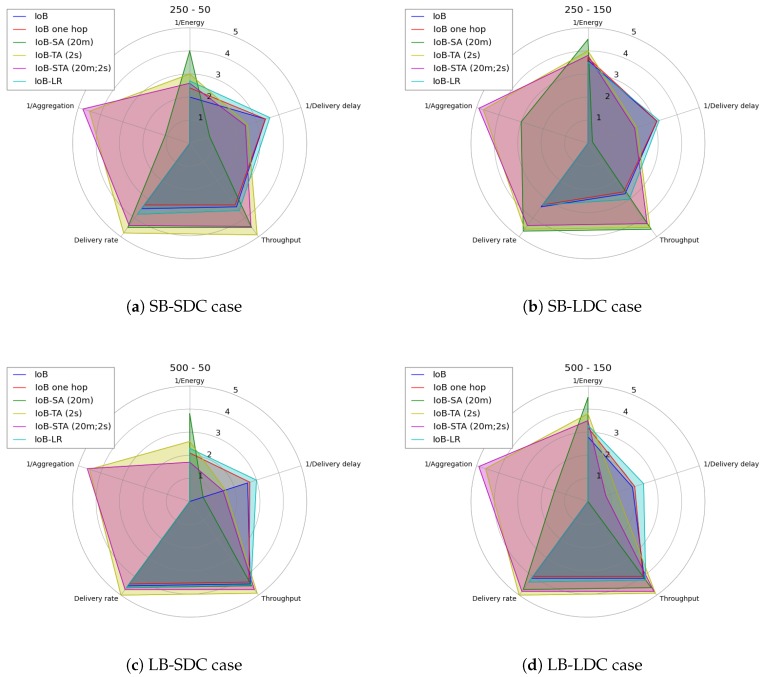
Performance comparison of IoB protocol variants.

In an urban application, the choice of which variant of IoB should be used depends on the sensed values. To understand the limitations of each variant of IoB, we depict in Figure 46 a radar schema summarizing their performances with respect to evaluated metrics. Here, we use a score between 1 and 5 illustrating that the higher value offers the best performance.

**Figure 47:**
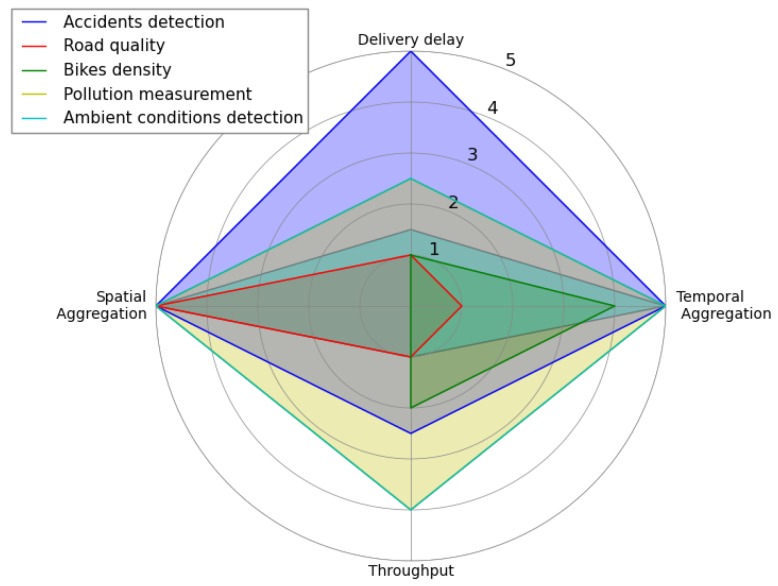
Performance comparison between five applications.

As mentioned above, each application needs to achieve better performances for some metrics than others. For that, we present, as an example in Figure 47, another radar schema that indicates the performances required for five proposed applications with regard to temporal aggregation, spatial aggregation, delivery delay and throughput. For example, accident detection application require an important delay to be announced as well as an important temporal and spatial aggregation since the time and place of an accident are major parameters in such application while it requires a moderate throughput. Considering the road quality application, it requires a low delivery delay to be transmitted, low throughput and temporal aggregation. The paramount parameter, in this application, is the road location.

## 9. Conclusions

This paper focuses on the application of Internet of Things (IoT) on real networks and in particular on connected bikes. We introduced the “Internet of Bikes”, IoB-DTN routing protocol being applied to mobile network IoT devices running a data collection application. First, we evaluated the impact of the variation of three parameters on the proposed protocol: buffer management policies, number of copies of a packet sprayed in the network and the transmission power value. Our simulation results show that GPP policy, limiting the number of copies sprayed in the network and using 10 mW as transmission power of sensors offer the best trade-off among simulated metrics. Next, we gave a detailed performance comparison between the multi-hop IoB-DTN protocol with a low-power wide-area network (LPWAN) technology, LoRa type with respect to two metrics: energy consumption and throughput. The obtained results show that using IoB-DTN protocol based on a multi-hop topology offers better throughput while using a long-range technology where there is only bike to bike station communication gives better performance on the energy consumption. Finally, we proposed an efficient solution for the multi-hop IoB-DTN protocol to cope with this result. Data aggregation approach is applied leading the combination of several data packets, according to some conditions, into a single packet. We proposed three variants of IoB-DTN based on this mechanism: IoB based on spatial aggregation (IoB-SA), IoB based on temporal aggregation (IoB-TA) and IoB based on spatiotemporal aggregation (IoB-STA). The simulation results showed that the three variants provide better performances with respect to many metrics, comparing to IoB-DTN without aggregation and a low-power long-range technology, LoRa type.

## Figures and Tables

**Table 1:** Simulated cases.

	Buffer Size	Duty Cycle (s)
SB-SDC	250	50
SB-LDC	250	150
LB-SDC	500	50
LB-LDC	500	150

**Table 2:** Simulation parameters.

Number of bikes	47
Number of bike stations	49
Packet generation time	Every second
Packet size	160 Bytes
Number of copies	8
Communication model	802.11p
Transmission power	10 mW
Simulation time	30 min

**Table 3:** Parameters used in our theoretical results.

	IoB	IoB-LR
**Model**	Datasheet Qualcomm AR6004	Semtech SX1272
**Tx**	237 mA	26 mA
**Rx**	66 mA	12 mA
**Packet duration**	213 μs	250–50: 0.2 s
		250–150: 0.6 s
		500–50: 0.1 s
		500–150: 0.3 s
**ACK duration**	213 μs	0.05 s
**Packet size**	160 Byte	250–50: 92 Byte
		250–150: 260 Byte
		500–50: 20 Byte
		500–150: 175 Byte

**Table 4:** Payload sizes (PS) of IoB-LR with different SF.

	Airtime (s)	PS (Byte)	PS (Byte)	PS (Byte)	PS (Byte)	PS (Byte)	PS (Byte)
		SF 7	SF 8	SF 9	SF 10	SF 11	SF 12
**250–50**	0.2	92	40	-	-	-	-
**250–150**	0.6	260	220	100	28	-	-
**500–50**	0.1	20	-	-	-	-	-
**500–150**	0.3	175	80	25	-	-	-
